# Synthesis, Characterization, and Dual Functional Properties of Coumarin-Based Hybrids for Biological and Optical Applications

**DOI:** 10.1021/acsomega.5c08426

**Published:** 2025-10-16

**Authors:** Juliana G. M. Lima, Jhonathan R. N. dos Santos, Luis M. G. Abegão, Leandro H. Zucolotto Cocca, Leonardo R. de Almeida, Hamilton B. Napolitano, Luciano Ribeiro, Luciana M. Ramos

**Affiliations:** † Laboratório de Química Medicinal e Síntese Orgânica, 271384 Universidade Estadual de Goiás, 75132-903, Anápolis Goiás, Brazil; ‡ Grupo de Química Teórica e Estrutural de Anápolis, 74391 Universidade Estadual de Goiás, 75132-903 Anápolis, Goiás, Brazil; § Departamento de Física, Universidade Federal de Sergipe, 49107-230, São Cristóvão Sergipe, Brazil; ∥ Grupo de Fotônica, Instituto de Física, 67824 Universidade Federal de Goiás, Goiânia 74690-900, Goiás, Brazil

## Abstract

Heterocyclic compounds incorporating nuclei of coumarin, benzimidazole, and benzothiazole exhibit significant biological and pharmacological properties. This study presents an efficient synthetic route for hybrid molecules integrating coumarin with either benzimidazole or benzothiazole under mild reaction conditions. The antibacterial and antioxidant properties of the synthesized compounds were systematically evaluated. The methodology successfully yielded 11 compounds in total, including six substituted coumarin–benzimidazole and five coumarin–benzothiazole derivatives, with yields ranging from 14 to 60%. Notably, derivatives **19** and **26** exhibited moderate antibacterial activity against *Staphylococcus aureus* at concentrations of 500 and 250 μg·mL^–1^, respectively. Derivatives **26** and **33** demonstrated strong antioxidant activity, with DPPH radical scavenging rates between 89 and 72%. Structure–activity relationship analysis revealed that substituents at position 7 of the coumarin nucleus significantly enhance antioxidant activity. Density functional theory calculations were performed to evaluate the compounds. Multivariate statistical methods, such as principal component analysis, identified molecular volume and bond order as key determinants of biological activity. Additionally, optical properties were explored, revealing promising photoluminescence characteristics with emission ranging from 446 to 502 nm. These findings suggest potential applications in theranostics and optoelectronics. This work highlights the importance of structural modifications in optimizing the biological and optical properties of coumarin-based hybrids, providing a foundation for future studies aimed at expanding their biomedical and technological applications.

## Graphical abstract

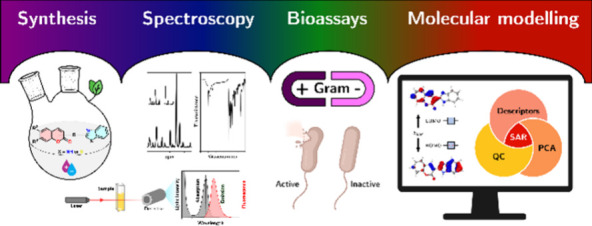

## 1 Introduction

Among heterocyclic compounds, two prominent nuclei are coumarins **1** and benzimidazoles/benzothiazoles **2** or **3** (Figure 1). These compounds exhibit a broad spectrum of biological activities and are present in various pharmaceuticals. The benzimidazole **2** and benzothiazole **3** nuclei are particularly notable for their key biological properties, which include antiviral,^1,2^ anti-inflammatory,^3^ antibacterial,^4^ antioxidant,^5^ antifungal,^6^ and antitumor activities.^7^ Benzimidazole-based drugs are widely utilized for antiparasitic,^8^ antihistamine, antihypertensive,^3,5^ antigastric ulcer,^7^ analgesic, and anti-inflammatory applications.^9^ Similarly, benzothiazole derivatives are commonly employed in fungicides, anticonvulsants, and herbicides.^10–12^

**Figure 1:**
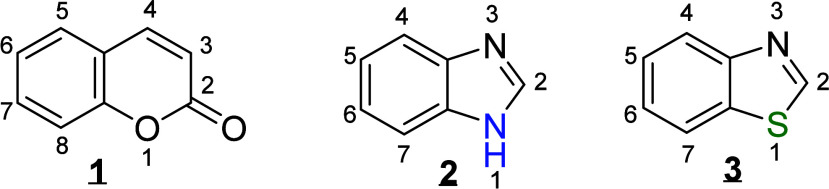
Structure of coumarin ( **1** ), benzimidazole ( **2** ), and benzothiazole ( **3** ) nuclei and their important positions for substitution.

Coumarins **1** exhibit a wide range of pharmacological properties, including antibiotic,^13^ anti-inflammatory,^14^ analgesic,^15,16^ anticoagulant,^17^ antiviral,^18^ antifungal,^19,20^ antimicrobial,^21,22^ and anticancer activities.^23^ Due to their inherent bioactivity, specific substituents can be introduced to the coumarin nucleus to enhance targeted properties.^24^ For example, the incorporation of aromatic groups such as benzimidazoles and benzothiazoles into coumarins represents a promising synthetic strategy, owing to their combined biological activities.^5,13,25,26^ In structure–activity relationship (SAR) studies, benzimidazole **2** and benzothiazole **3** cores substituted at positions 1, 2, 5, and 6 with heterocyclic, alkyl, or aryl groups demonstrated potential for anti-HIV, antituberculosis,^27^ and antitumor activities.^28^ Similarly, substitutions at positions 3, 4, or 7 of the coumarin core have been shown to enhance cytotoxic and antimicrobial effects.^29–31^

In recent years, the synthesis of hybrid molecules incorporating multiple bioactive components has emerged as a prominent strategy in medicinal chemistry.^32–34^ These molecules are designed to target multiple sites simultaneously, potentially improving their individual biological activities.^8,13^ The synthesis of hybrid compounds involves integrating diverse functional groups to produce novel substances with improved or synergistic properties.^13^ This hybrid approach to the synthesis of coumarin-benzimidazole/benzothiazole derivatives represents a promising strategy for discovering compounds with significant pharmacological potential. For example, aromatic groups attached at position 3 of the coumarin nucleus and position 2 of the benzimidazole or benzothiazole nuclei have been shown to enhance the antimicrobial activity of the resulting hybrid compounds.^13^ Typically, hybrids **6** are synthesized by reacting *o*-phenylenediamine **5** with structurally diverse coumarins **4** , typically under catalytic conditions and thermal activation (Scheme 1).^35^

**Scheme 1:**
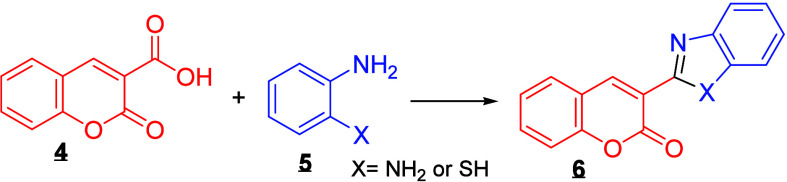
. General Synthesis of Coumarin-Benzimidazole/Benzothiazole Hybrids from Coumarin-3-carboxylic Acid

In the pursuit of efficient synthetic strategies, numerous procedures have been explored and documented in the literature. Among these, the use of ionic liquids (ILs) has gained significant attention due to their advantages, including ease of separation, potential for reuse, nontoxicity, and alignment with green chemistry principles.^36,37^ SAR studies involve theoretical investigations, often employing computational calculations, to elucidate the effects of a compound’s chemical structure on its interaction with biological receptors.^38^ Inspired by the intriguing pharmacological properties of these compounds, this work presents methods to synthesize hybrid derivatives of coumarin-benzimidazole/benzothiazole using acidic ionic liquids as catalysts. The study focuses on evaluating the biological activities of these hybrids and exploring their structure–activity relationships, yielding promising results. To correlate computational results with biological and antioxidant properties, quantum chemical calculations were performed using density functional theory (DFT),^39^ and the resulting theoretical descriptors were analyzed using multivariate statistical methods, notably principal component analysis (PCA).^40^

In addition, coumarin-based fluorophores have been developed, exhibiting far-red/near-infrared (NIR) emission, large Stokes shifts, high photostability, excellent brightness, and good cell membrane permeability, making them ideal for cell imaging.^41^ In particular, C6 substituted derivatives demonstrate strong intramolecular charge transfer, remarkable solvatochromism (536–714 nm), and multistimuli-responsive characteristics, positioning them as promising candidates for advanced fluorescence-based applications.^42^ The optical properties of the compounds were investigated using ultraviolet–visible (UV–vis) absorbance and fluorescence emission spectroscopy. These properties are influenced by structural factors such as conjugation, electron-donating/withdrawing groups, and heterocyclic moieties.^41,42^ The study highlights their potential for applications in fluorescence microscopy,^43^ theranostics, organic light-emitting diodes (OLEDs), and nonlinear optical technologies.^44^ By correlating optical properties with molecular structure, this work enhances our understanding of photophysical behavior, supporting the exploration of coumarin-based hybrids as promising candidates for photonic devices or emissive bioprobes.

## 2 Experimental and Computational Procedures

### 2.1 Synthesis and Spectroscopic Analysis

A series of coumarin derivatives with diverse substituents was synthesized for this study. In a sealed tube, 1 equiv of coumarin was combined with 3 equiv of *o*-phenylenediamine or 2-aminothiophenol, 40 mol % of methyl-acetyl-imidazole (MAI.Cl) as catalyst, and 3 mL of ethanol. The mixture was heated to reflux under constant stirring at 80 °C for 24 h or until the reaction reached completion. After cooling, the product was isolated by precipitation in ice-cold ethanol, vacuum filtered and recrystallized from hot ethanol to obtain a yellow solid, with yields ranging from 14 to 60%. Additional details regarding the spectroscopic apparatus and methodology are provided in the Supporting Information (SM1).

### 2.2 Antibacterial Activity

The antibacterial activity of the synthesized compounds was evaluated using a microdilution broth method to determine the minimum inhibitory concentration (MIC) and minimum bactericidal concentration (MBC). The assay followed the guidelines established by the Clinical and Laboratory Standards Institute (CLSI)^45^ and was carried out in a 96-well microplate format. Standard strains from the American Type Culture Collection (ATCC) were used: Gram-positive *Staphylococcus aureus* (ATCC 25923) and Gram-negative *Escherichia coli* (ATCC 25312). Bacterial inocula were prepared by suspending bacteria in sterile saline (0.9% NaCl) and adjusting turbidity to match a 0.5 McFarland standard, as determined by spectrophotometric measurement at 625 nm. The suspension was then diluted 1:10 in saline to achieve a final concentration of 10^7^ CFU mL^–1^. For MIC determination, a sterile 96-well U-bottom microplate was used. The compounds were solubilized in 5% dimethyl sulfoxide (DMSO) and serially diluted in Mueller-Hinton broth (MH) to obtain concentrations of 2000, 1000, 500, 250, 125, 62.5, and 31.2 μg·mL^–1^. The same procedure was applied to the standard antibiotic chloramphenicol (Inlab) at concentrations of 64, 32, 16, 8, 4, 2, and 1 μg·mL^–1^ to validate the assay. The control wells included 5% DMSO, medium sterility, and sample sterility. All assays were performed in triplicate.

In each well, 100 μL of the prepared compound solutions, 100 μL of MH broth, and 7 μL of the bacterial inoculum were added. The microplates were incubated at 35 °C for 24 h. After incubation, the plates were visually inspected to determine the lowest concentration that inhibited bacterial growth (MIC). For MBC determination, 100 μL aliquots from wells that did not show bacterial growth were plated onto nutrient agar plates and incubated at 35 °C for 24 h. After incubation, the plates were inspected to identify the lowest concentration that completely prevented bacterial growth.

### 2.3 Antioxidant Activity

The antioxidant activity of the compounds synthesized was evaluated using the 2,2-diphenyl-2-picrylhydrazyl (DPPH) radical scavenging assay with adaptations.^46,47^ The assay was carried out in a 96-well plate, and absorbance readings were taken using a UV–vis spectrophotometer at 517 nm, with methanol as the calibration solvent. A calibration curve for DPPH was prepared using a DPPH solution in methanol at concentrations of 50, 25, 12.5, and 6.25 μM, kept in the dark. The synthesized compounds were dissolved in DMSO and diluted in methanol to achieve concentrations of 200, 100, 50, 25, 12.5, and 6.25 μM. The same procedure was applied to the standard antioxidant Quercetin (Sigma-Aldrich) at these concentrations to validate the assay. Antioxidant viability controls included DMSO in methanol, a 0.1 mM DPPH methanolic solution and the standard antioxidant. All assays were performed in triplicate.

For each well, 50 μL of the test solutions (at various concentrations) or viability controls was added to 50 μL of the DPPH solution. The plate was kept in the dark for 30 min before absorbance measurements were taken using an ELISA reader. Antioxidant activity was expressed as the percentage of inhibition (%AA), calculated using the formula:

$$\%\mathrm{AA}=\left[\frac{{\mathrm{Abs}}_{\mathrm{control}}-{\mathrm{Abs}}_{\mathrm{sample}}}{{\mathrm{Abs}}_{\mathrm{control}}}\right]100$$ 1

The amount of sample required to inhibit the radical by 50% (IC_50_) was determined using the linear equation derived from the plot of concentration versus percentage of inhibition. The results were statistically analyzed and compounds showing IC_50_ values comparable to the standard were classified as active or inactive according to their performance.

### 2.4 Molecular Modeling

A conformational analysis of the synthesized compounds was performed using the molecular mechanics method with the MM^+^ force field.^48^ Geometry optimization was carried out using Gaussian 16 software^49^ with the M06-2X^50^ functional and the 6-311++G­(d,p) basis set.^51,52^ The global minimum geometries for each structure of compounds were confirmed by the absence of imaginary harmonic frequencies in any normal modes through DFT calculations. The global minimum structures of the gas systems of compounds are given in Supporting Information (SM1, Tables S1–S11).

The following molecular descriptors were calculated: the energies of the highest occupied molecular orbital (HOMO) and the lowest unoccupied molecular orbital (LUMO), electronic gap energy (*E*_GAP_ = *E*_LUMO_ – *E*_HOMO_), dipole moment, ionization energy (calculated as the negative of the HOMO energy), electron affinity (calculated as the negative of the LUMO energy), hardness, electronic chemical potential, electrophilicity index, surface area, volume, refractivity, polarizability, and Log P.^53^ Additionally, interatomic distances, bond angles, torsions (dihedral angles), natural bond orders, and atomic charges derived from the electrostatic potential were obtained using the CHELPG method.^54^ A table listing the calculated molecular descriptors is available in Supporting Information (SM2).

### 2.5 Optical Properties

UV–Vis absorption and fluorescence emission spectra were recorded in DMSO and chloroform for selected compounds to explore their photoluminescence characteristics and potential applications in fluorescence-based imaging and optoelectronics. The absorbance and fluorescence emission spectra of the investigated compounds were measured using carefully prepared solutions to ensure reliable data. Solutions with concentrations of approximately 10^–5^ M for absorbance measurements and 10^–6^ M for fluorescence measurements were prepared in DMSO for most compounds. However, compounds **26** and **30** were dissolved in chloroform due to better solubility in this solvent. The chosen solvents not only provided optimal solubility but also prevented the formation of aggregates. The samples were placed in standard 10 mm thick fused quartz cuvettes for all measurements. Absorbance spectra were recorded using a UV–Vis spectrophotometer (Global Analyzer, GTA-36), while fluorescence emission spectra were acquired using a fluorescence spectrophotometer (HORIBA-Jobin Yvon, Fluorolog FL3–221), with excitation performed in the lowest energy band.

## 3 Results and Discussion

### 3.1 Synthesis and Spectroscopic Analysis

Coumarins were efficiently synthesized following established procedures^24^ and further modified to introduce structural diversity through the incorporation of various substituents. Optimal reaction conditions were identified by systematically varying key parameters, including the catalyst, solvent, temperature, reaction time, and reagent ratios. The derivatives were synthesized via reflux in a sealed Schlenk tube for 24 h, reacting six distinct coumarins with either *o*-phenylenediamine, 2-aminothiophenol, or 4-methyl-*o*-phenylenediamine derivative **8** , using MAI.Cl as catalyst. This ionic liquid catalyst was prepared according to previously reported protocols.^55^ Scheme 2 shows the general synthetic approach for the coumarin-based hybrids.

**Scheme 2:**
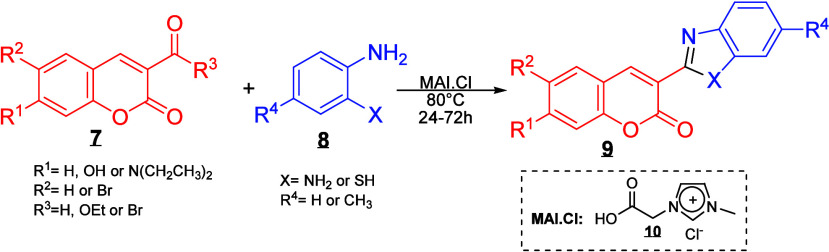
. Synthetic Route to Coumarin-Based Hybrids

In an initial series of experiments, the reaction condition was optimized by evaluating various catalysts, including *p*-toluenesulfonic acid (*p*-TSOH), ferric chloride (FeCl_3_), 1-methyl-3-(3-sulfopropyl) imidazolium (MSI), and methyl-acetyl-imidazole (MAI.Cl). The influence of different solvents — ethanol (EtOH), water (H_2_O), and tetrahydrofuran (THF) — was also investigated. Additionally, the effects of temperature and reaction time on yield were systematically examined. The best results were achieved using MAI.Cl as the catalyst in ethanol with stirring at 80 °C for 24 h, providing a yield of 60% for the desired product (see Table 1).

**Table 1:** Optimization of Reaction Conditions for Hybrid Synthesis with Ionic Liquid in Catalysis

entry	catalyst^a^	solvent	time (h)	yields^b^ (%)
1	*p*-TSOH *	EtOH	2	12
2	FeCl_3_*	EtOH	2	trace
3	MSI*	EtOH	2	trace
4	MAI.Cl*	EtOH	2	24
5	MAI.Cl**	EtOH	4	42
6	MAI.Cl**	BMI.BF_4_	4	15
7	MAI.Cl**	DMF	4	trace
8	MAI.Cl**	EtOH	12	42
9	MAI.Cl**	EtOH	24	60

a Reactions Conditions: 3 mmol *o*-phenylenediamine, 1 mmol coumarin, 80 °C and 20 mol %* or 40 mol % catalyst.

b Isolated yields.

The imidazolium groups in ionic liquids, such as MAI.Cl, plays a crucial role in facilitating reactions by enhancing reactivity and promoting the formation of the desired products. In the proposed mechanism for the formation of the hybrid compound 3-(1*H*-benzo­[*d*]­imidazol-2-yl)-2*H*-chromene-2-one **19** (see Scheme 3), the reaction starts with the protonation of coumarin **11** via the acidic hydrogen of catalyst **10** . This protonation activates the carbonyl group of the coumarin, increasing its electrophilicity and forming intermediate **12** . Subsequently, the nucleophilic addition of *o*-phenylenediamine **13** to the activated carbonyl group leads to the formation of intermediate **14** , which undergoes prototropism **15** , followed by the elimination of ethanol. The next step involves a second nucleophilic attack by the NH_2_ group on the carbonyl group, which is facilitated by acid catalysis, leading to intramolecular cyclization **16** . This step results in the elimination of water **18** and the formation of the final product **19** .

**Scheme 3:**
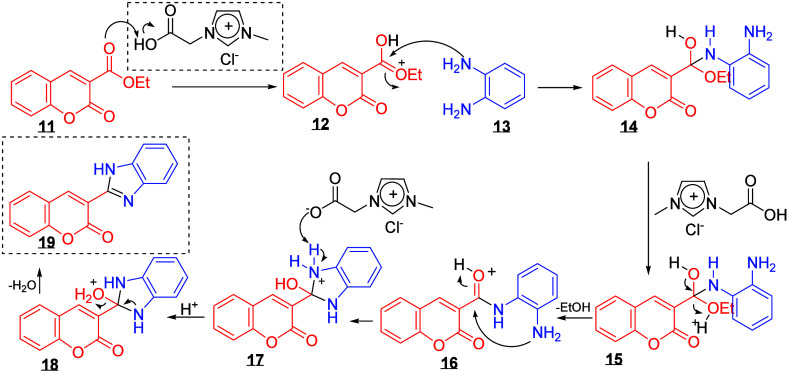
Proposed Mechanism for the Synthesis of **19**

Having established a general protocol for synthesizing coumarin-benzimidazole/benzothiazole hybrids, we employed various starting coumarins to produce 11 derivatives (see Table 2), achieving isolated yields ranging from 14 to 60%. This variation can be attributed to differences in the reactivity of the starting materials and the electronic effects of the substituents. Using MAI.Cl as a catalyst was effective in promoting the formation of the desired hybrids, likely due to its ability to stabilize intermediates and enhance reaction rates. The relatively low yields observed in the synthesis can be attributed to the low nucleophilicity of *o*-phenylenediamine (p*K*_a_ ∼ 4.47) and 2-aminothiophenol (p*K*_a_ ∼ 4.84), combined with the low electrophilic character of Ethyl-2-oxo-2*H*-chromene-3-carboxylate. This approach is characterized by its sustainability and the use of ionic liquids as catalysts in the synthesis of benzimidazoles and benzothiazoles. In contrast to conventional protocols that utilize POCl_3_,^35^ which activate ester-linked groups but generate considerable waste, the present strategy offers a more environmentally friendly and efficient alternative. These factors necessitate the use of a catalytic medium to facilitate the reaction. Typically, the rate-limiting step is the nucleophilic attack on the ester carbonyl, which is hindered by the low basicity of the amines and thereby compromising the reaction efficiency. All products were characterized using spectroscopic techniques, including FTIR (KBr), ^1^H and ^13^C­{^1^H} NMR, with detailed data provided in the Supporting Information (SM1, and Figures S1–S35).

**Table 2:**
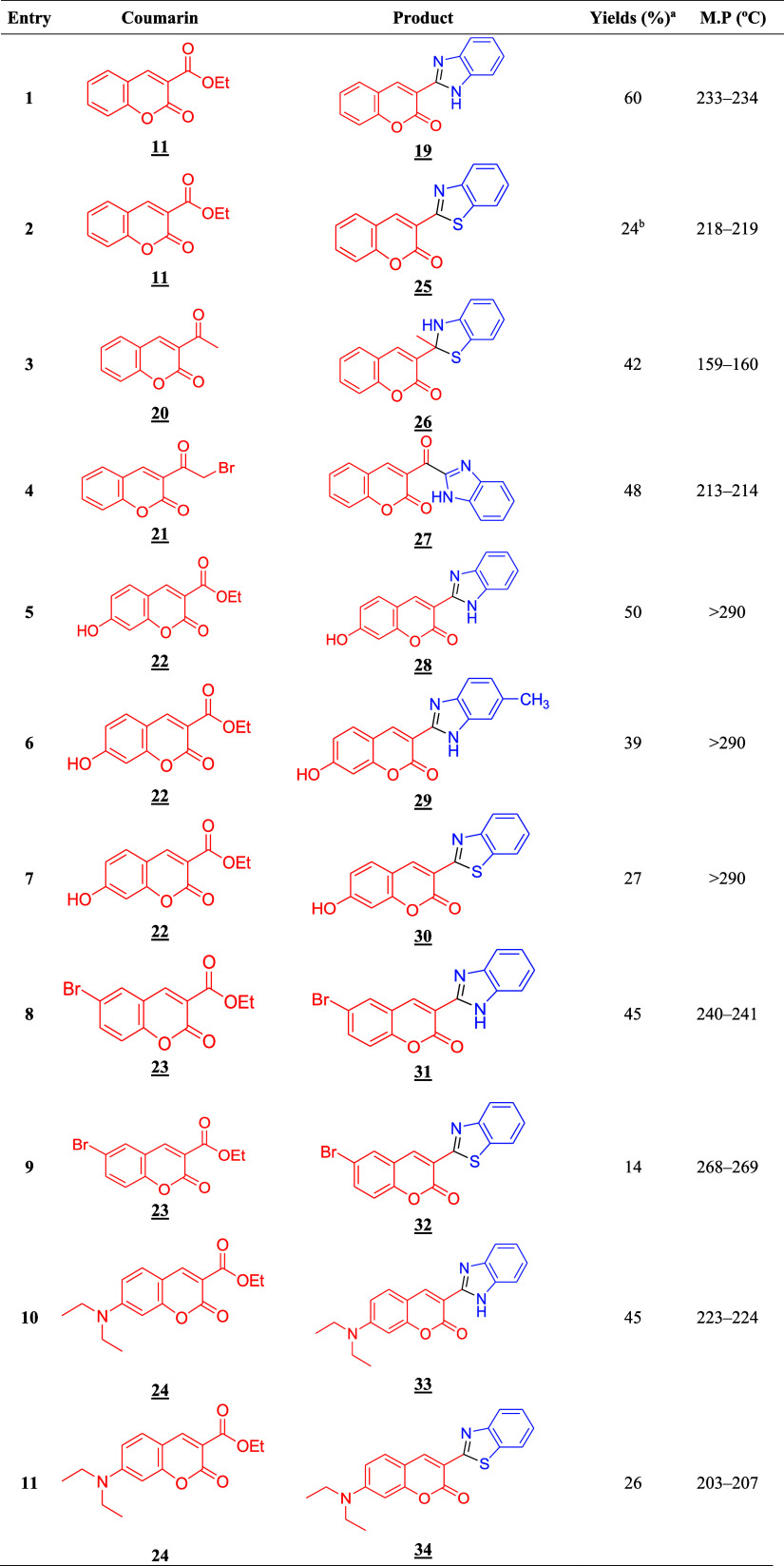
List of Synthesized Hybrid Derivatives

a Conditions: 1 mmol coumarin, 3 mmol *o*-phenylenediamine or 2-aminothiophenol or 4-methyl-*o*-phenylenediamine derivative, 40 mol % MAI.Cl, 3 mL EtOH, 80 °C, 24 h.

b Product obtained in 72 h of reaction.

Conformational analysis of a given molecule reveals insights into its three-dimensional structure, including bond lengths and angles, as well as the orientation of functional groups. This analysis provides valuable information on how substitution patterns influence stereochemistry and biological activity, and on the relationship between molecular structure and the physical properties of the same compound. Structures **26** and **27** exhibited distinct conformational arrangements compared to other derivatives, primarily due to the groups linked at position 2 of the benzimidazole ring. In contrast, the remaining molecules displayed similar conformations, confirming that the presence of benzimidazole/benzothiazole and coumaric rings results in a consistent planar structure, independent of substitutions on the aromatic systems. The coplanar arrangement of the coumaric and benzimidazole/benzothiazole rings creates a rigid planar system, resulting in uniform conformations across the derivatives.

These findings underscore the significance of catalyst and solvent selection in achieving more environmentally friendly and efficient synthetic routes for coumarin-benzimidazole/benzothiazole hybrids. The optimized conditions not only enhanced the yield but also demonstrated the potential for scalability and practical application in the synthesis of these compounds. The structural diversity achieved through the synthetic strategy permitted a comprehensive exploration of the relationship between molecular structure and functional properties. The incorporation of electron-donating and electron-withdrawing groups on the coumarin and benzimidazole/benzothiazole moieties provided valuable insights into the modulation of optical and biological activities, as discussed in subsequent sections.

### 3.2 Antibacterial and Antioxidant Activities

The antibacterial assays revealed that certain derivatives exhibited notable sensitivity against *S. aureus*. Specifically, compounds **19** and **26** demonstrated MIC of 500 and 250 μg·mL^–1^, respectively, indicating moderate antibacterial activity. In contrast, the other tested compounds did not inhibit the growth of *S. aureus*, and none of the compounds showed effectiveness against *E. coli*. Notably, compound **26** was able to eliminate *S. aureus* at concentrations up to 250 μg·mL^–1^, thereby highlighting its moderate bactericidal potential. Moreover, the presence of electron-donating groups at position 1 and bulky lipophilic groups at position 2 of the benzimidazole nucleus appeared to enhance antimicrobial activity.

The standard antioxidant quercetin exhibited antioxidant activity ranging from 94 to 70%. Among the synthesized derivatives, compounds **26** and **33** demonstrated superior radical scavenging potential, with antioxidant activities between 89 and 72%, approaching the values of the standard and thus considered highly active antioxidants. Compounds **19** , **25** and **27** showed DPPH radical reduction levels between 65 and 71%. These results suggest that the presence of coumarin-benzimidazole or coumarin-benzothiazole nuclei alone does not confer high antioxidant activity. Instead, the antioxidant potential of the hybrid compounds is influenced by the substituents and their positions within the molecular structure. For instance, derivatives **28** , **29** and **30** exhibited moderate antioxidant activity, ranging from 67 to 77%. It was observed that substituents such as amines, sulfur, hydroxyls, and methyl groups contributed to increased antioxidant activity. These groups facilitate the stabilization of the DPPH radical, thereby enhancing the antioxidant action.

### 3.3 Molecular Modeling Study

Figure 2 illustrates the trivial molecular structure numbered among the synthesized compounds listed in Table 2. Principal component analysis was employed to evaluate various combinations of molecular descriptors using the R Statistical Software (v4.4.2).^56^ The combination of bond order (B.O.29) between carbon 19 and the elements at position 29, the intermolecular distance between these atoms [D31-R­(19,29)], the van der Waals molecular volume, and the bond order (B.O.30) between carbon 20 and the elements at position 30 effectively distinguished active from inactive compounds. Table 3 lists these descriptors, highlighting the numbering of compounds that follow the trivial geometry, see in Figure 2 except for compounds **26** and **27** .

**Figure 2:**
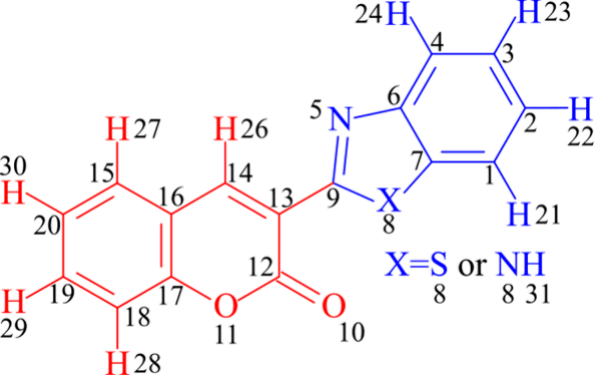
Representation of the numbered molecular structure of hybrid coumarin-benzimidazole.

**Table 3:** Values of Molecular Descriptors Calculated Using the DFT Method at the M06-2X/6-311++G­(d,p) Level and Selected by PCA for Antioxidant Activity Evaluation^a^

compounds		B.O.29	D31-R(19,29) [Å]	volume [Å^3^]	B.O. 30
19	inactive	1.3830	1.0832	734.025	0.9227
25	inactive	1.3824	1.0833	749.750	0.9229
28	active	1.3215	1.3531	808.185	1.0358
29	active	1.3213	1.3527	755.346	1.0370
30	active	1.3201	1.3526	771.214	1.0378
31	active	1.3560	1.0822	796.936	0.9136
32	active	1.3556	1.0822	812.484	0.9137
33	active	1.2615	1.3760	960.000	1.1447
34	active	1.2601	1.3754	974.948	1.1469

a B.O.29: bond order between elements 19 and 29; D31-R­(19,29): distances between elements 19 and 29; Volume: the *van der Waals* molecular volume; B.O.30: bond order between elements 20 and 30.

As illustrated in Figure 3a, the initial two principal components, designated PC1 and PC2, are represented by a vertical line distinguishing active (blue) from inactive (red) derivatives. For clarity, it should be noted that the highlighted figure corresponds exclusively to the derivatives enumerated in Table 2. PC1 explained 87.04% of the variability, effectively separating active from inactive compounds. This finding suggests that the primary discriminating factors between active and inactive compounds are strongly associated with the variables contributing to PC1. PC2 contributed to a lesser extent to the separation, and thus the discussion will focus on PC1.

**Figure 3:**
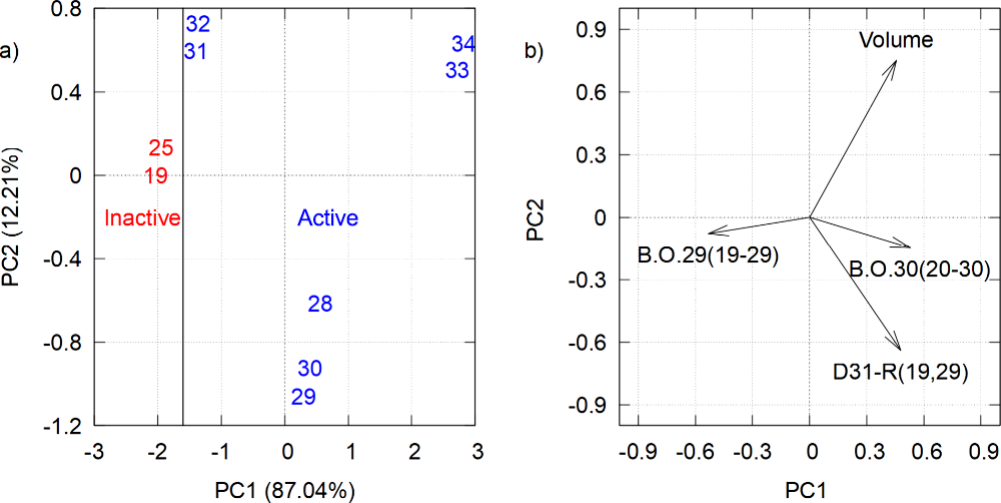
Principal components of active and inactive molecules for antioxidant activity.

Figure 3b illustrates the relationship between the selected descriptors and the principal components. The vectors indicate the direction and magnitude of each variable’s influence. The projection of the B.O.29 bond order vector is negative along PC1, suggesting that higher values of this variable are associated with inactive compounds. For instance, inactive compounds **19** and **25** exhibit higher B.O.29 values, which may inhibit antioxidant activity (see Table 3). Thus, reducing this bond order could be an effective strategy to improve activity. Conversely, the Volume descriptor demonstrates a positive correlation with PC1, indicating that compounds with larger molecular volumes tend to exhibit higher antioxidant activity. The active compounds (positioned on the right side of PC1) demonstrate higher volumes, suggesting a direct correlation with their molecular interaction capability. This hypothesis is further supported by the presence of bromine, a bulk atom, in compounds **31** and **32** . Therefore, increasing molecular volume has emerged as a prospective synthetic strategy to enhance antioxidant activity.

The B.O.30 vector is positively directed along PC1, indicating that a higher bond order in this region is a characteristic of active compounds. It is hypothesized that the adjustment of this bond order could result in the optimization of future compounds. Finally, the D31-R­(19,29) vector is positively positioned along PC1, suggesting that larger distances between atoms 19 and 29 favor antioxidant activity. It can be posited that smaller distances (negative PC1 values) may be indicative of more compact structures, which may be less favorable for antioxidant activity.

The Pearson correlation matrix of the descriptors listed in Table 3 is presented in Table 4, obtained using the R software. The correlation values are used to measure the relationship between variables; values close to +1 indicate a positive linear correlation, while values close to −1 indicate a negative or inverse correlation. A strong negative correlation of −0.966 between B.O.29 and B.O.30 suggests a significant inverse relationship, indicating structural compensation between these bonds, likely due to electronic or steric rearrangements. This finding is consistent with the vector projections depicted in Figure 3b.

**Table 4:** Pearson Correlation Coefficients between Geometric and Electronic Descriptors^a^

	B.O.29	D31-R(19,29)	volume	B.O.30
**B.O.29**	1	–0.863	–0.871	–0.966
D31-R(19,29)	–0.863	1	0.526	0.921
**volume**	–0.871	0.526	1	0.784
**B.O.30**	–0.966	0.921	0.784	1

a B.O.29: bond order between elements 19 and 29; D-31-R­(19,29): distances between elements 19 and 29; Volume: the van der Waals molecular volume; B.O.30: bond order between elements 20 and 30.

The correlation of −0.863 between B.O.29 and D31-R­(19,29) is also strong and negative, indicating that an increase in B.O.29 bond order is associated with a decrease in the distance D31-R­(19,29). This finding is consistent with the typical shortening effect of stronger bonds. In addition, the strong negative correlation of −0.872 between B.O.29 and molecular Volume suggests that compounds with a higher B.O.29 bond order tend to have a smaller molecular volume, implying that stronger bonds may limit molecular expansion. On the other hand, the high positive correlation of 0.921 between D31-R­(19,29) and B.O.30 indicates that an increase in the distance D31-R­(19,29) is associated with a strengthening of the B.O.30 bond. This suggests that a greater separation between atoms 19 and 29 may lead to electronic redistribution, thereby enhancing the strength of the 20–30 bond.

As illustrated in Figure 4, the frontier molecular orbital energies (HOMO, LUMO) and the corresponding HOMO–LUMO gaps (*E*_GAP_) are presented for coumarin derivatives (19 **,** 25 **,** 26 **,** 27 **,** 28 **,** 29 **,** 30 **,** 31 **,** 32 **,** 33 **,** and **34**). The *E*_GAP_ values range from 512.1 to 590.8 kJ·mol^–1^, thus demonstrating significant tunability of electronic properties through structural modifications. These energy gaps are critical for determining charge transfer characteristics, optical properties, and chemical reactivity in coumarin-based systems. Compounds **33** (512.9 kJ·mol^–1^) and **34** (512.1 kJ·mol^–1^) exhibit the smallest band gaps, a phenomenon that correlates with enhanced intramolecular charge transfer (ICT) and experimentally observed red-shifted in both absorption and emission spectra (see UV–Vis data in Table 5). In contrast, compound **27** (590.7 kJ·mol^–1^) exhibits the largest band gap, suggesting enhanced kinetic stability and a concomitant blueshifted optical profile.

**Figure 4:**
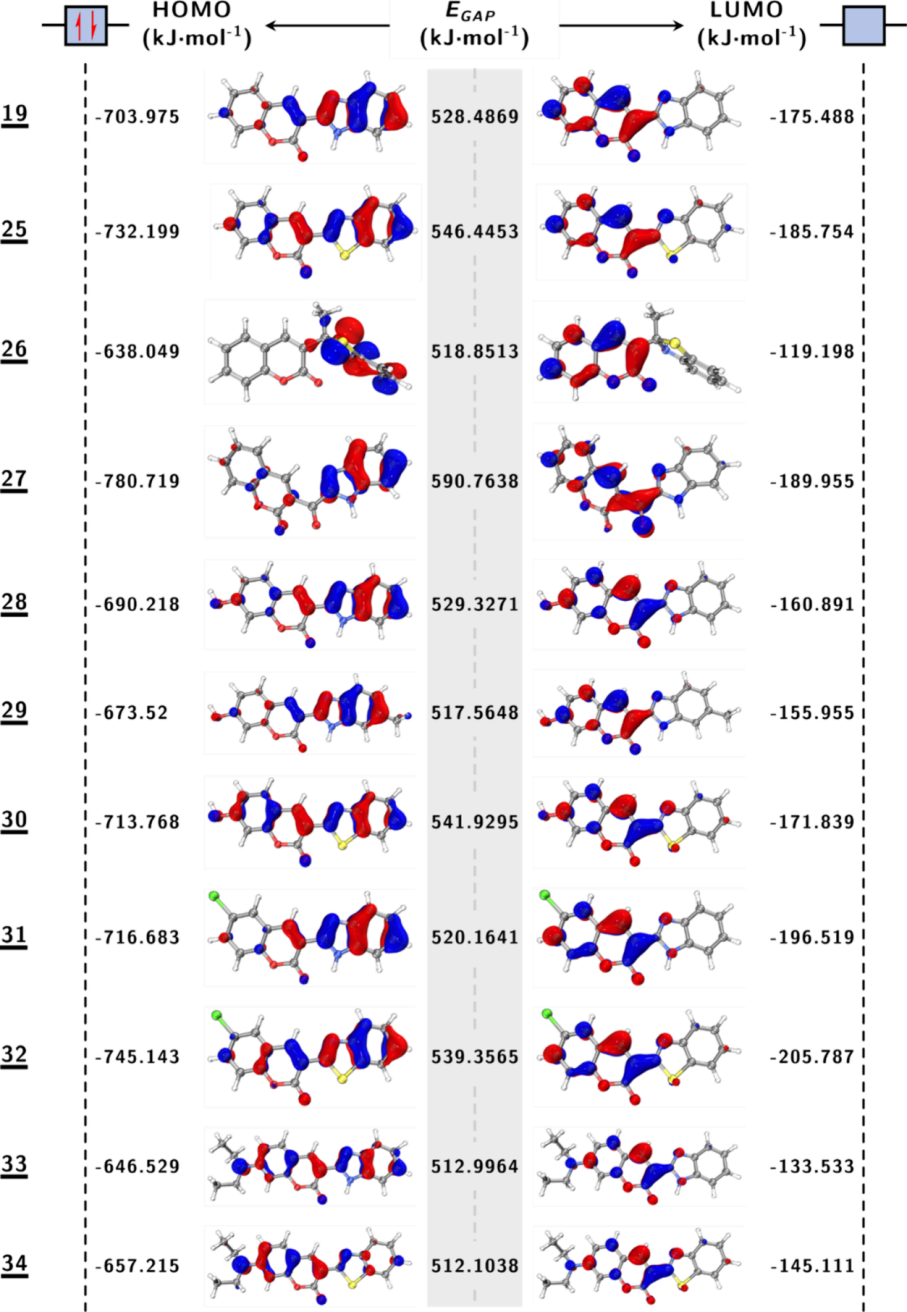
Frontier molecular orbitals for 11 compounds: HOMO (left), HOMO–LUMO energy gap (*E*_GAP_, kJ·mol^–^^1^, center), and LUMO (right), computed at the M06-2X/6-311++G­(d,p) level of theory.

**Table 5:** Spectral Positions of the Investigated Compounds for Maximum Absorbance (λ_ABS_^max^) and Fluorescence Emission (λ_FL_^max^) in DMSO, Except for Compounds **26** and **30** , Which Were Dissolved in Chloroform^a^

compound	**λ**_ABS_^max^ [**nm**]	**λ**_FL_^max^ [**nm**]	**Δλ** [**nm**]
**19**	297	476	179
**25**	365	486	121
**26**	290	466	176
**27**	298	494	196
**28**	318	479	161
**29**	388	502	114
**30**	395	477	82
**31**	357	446	143
**32**	427	484	57
**33**	429	486	57
**34**	423	485	62

a The Stokes shift (Δλ = λ_FL_^max^ – λ_ABS_^max^) is also included.

### 3.4 Optical Properties

The fluorescence emission spectral positions of the investigated compounds span a wide range, from 446 nm (compound **31** ) to 502 nm (compound **29** ), demonstrating their potential versatility in photoluminescence-based applications. These applications include fluorescence microscopy,^57^ theranostics for drug delivery and photodynamic therapy,^58^ and organic light-emitting diodes (OLEDs) for display technologies.^59^ This broad spectral range is influenced by structural features such as the degree of conjugation, electron-donating or withdrawing substituents, and the nature of the heterocyclic moiety attached to the coumarin core.

Compounds with electron-donating hydroxyl groups, such as **28** , **29** and **30** , exhibit intermediate spectral positions (318, 388, and 395 nm, respectively), as the hydroxyl group enhances π-electron delocalization but is not sufficient to achieve the most significant redshifts. In contrast, the most red-shifted compounds are **32** , **33** and **34** , with absorbance maxima at 427, 429, and 423 nm, respectively. These redshifts reflect the extensive conjugation in their thiazole-based structures.

On the other hand, compounds containing benzotriazole moieties, such as **19** , **27** , **29** , and **31** , show intermediate absorbance maxima, as the benzotriazole acts as an electron acceptor, contributing to ICT but not extending conjugation as extensively as thiazole. Finally, compound **26** , with its short maximum absorbance at 290 nm, exhibits a significantly lower degree of conjugation or resonance stabilization than the other compounds. Moreover, the structural similarity of the benzimidazole and benzothiazole nuclei to the imidazole and oxazole cores, which are well-known for their capability to enable two-photon absorption-induced fluorescence,^43,60^ suggests that the investigated compounds may exhibit nonlinear optical properties. This makes them promising candidates for advanced applications in nonlinear optical spectroscopy^61^ and photonic technologies. These structural features collectively explain the observed spectral shifts in maximum absorbance across the series, reported in Table 5 and depicted in the Supporting Information (SM1, Figures S36–S46).

## 4 Conclusions

Eleven derivatives were synthesized, with yields ranging from 14 to 60%, and their structures were confirmed through spectroscopic methods. The benzimidazole/benzothiazole moiety, when directly linked to the coumaric ring forms a rigid planar system, with the two aromatic classes aligned in the same plane, contributing to the stability and electronic properties of the compounds. In terms of antimicrobial activity, promising outcomes were noted for compounds **19** and **26** , which exhibited moderate antibacterial activity against *S. aureus*. This activity can be attributed to the presence of bulky electronic and lipophilic groups within their structures. However, none of the compounds demonstrated efficacy against *E. coli*. Regarding antioxidant activity, compounds **26** and **33** exhibited superior free radical scavenging potential, with activities ranging from 89 to 72%, approaching the performance of the standard antioxidant quercetin. The presence of functional groups such as amines, hydroxyls, and sulfur atoms was found to enhance antioxidant activity by facilitating the stabilization of free radicals.

A theoretical study of the structure–activity relationship was conducted using molecular descriptors obtained via DFT, analyzed employing PCA and Pearson correlation. It provided a detailed evaluation of the geometric and electronic descriptors of the hybrid coumarin-benzimidazole/benzothiazole compounds. The results in this study revealed that molecular expansion (geometric descriptors) and the optimization of chemical bonds (electronic descriptors) play complementary roles in determining antioxidant activity. For instance, compounds with larger molecular volumes exhibited higher antioxidant potential. The strong negative correlation between bond orders B.O.29 and B.O.30, as well as the positive correlation between molecular volume and antioxidant activity, provided valuable insights for designing more effective compounds.

The optical properties of the compounds, with fluorescence emission spectral positions ranging from 446 to 502 nm, suggest potential applications in fluorescence-based imaging, theranostics, and organic electronic materials such as OLEDs. The structural similarity of benzimidazole and benzothiazole nuclei to imidazole and oxazole cores further indicates potential for nonlinear optical properties, making these compounds promising candidates for advanced photonic technologies.

In conclusion, this study highlights the significance of structural modifications in tuning the biological and optical properties of coumarin-benzimidazole/benzothiazole hybrids. Future studies may concentrate on extending the synthetic scope, optimizing molecular descriptors for enhanced activity, and further evaluating their biomedical and photonic applications.

## Supplementary Material





## Notes

FTIR, ^1^H NMR, and ^13^C­{^1^H} NMR spectra of synthesized compounds; optical data from spectroscopy of absorbance and fluorescence emission spectra of synthesized compounds; and optimized Cartesian coordinates (Å) of compounds (PDF)
Molecular descriptors used in PCA analysis (XLSX)

## ⊥

J.R.N.d.S.: Laboratório de Química Medicinal e Síntese Orgânica, Universidade Estadual de Goiás, 75132–903, Anápolis, Goiás, Brazil. Current address: Instituto de Química, Universidade Federal de São Carlos, São Paulo, SP, Brazil
