# Supplementary material for: Synthesis, Characterization, and Dual Functional Properties of Coumarin-Based Hybrids for Biological and Optical Applications
Source: ACS Omega. 2025 Oct 16;10(42):50600–10. doi: 10.1021/acsomega.5c08426 (PMC12573153; doi:10.1021/acsomega.5c08426)
Supplement: Supplementary file 1 [file ao5c08426_si_001.pdf]

Supplementary Material

Synthesis, Characterization and Dual Functional Properties of Coumarin-based Hybrids for Biological and Optical Applications

Juliana G. M. Lima[a], Jhonathan R. N. dos Santos[a], Luis M. G. Abegão[c], Leandro H. Zucolotto Cocca[d], Leonardo R. de Almeida[b], Hamilton B. Napolitano[b], Luciano Ribeiro[b*] and Luciana M. Ramos[a*].

[a] Laboratório de Química Medicinal e Síntese Orgânica, Universidade Estadual de Goiás, 75132-903, Anápolis, Goiás, Brazil.

[b] Grupo de Química Teórica e Estrutural de Anápolis, Universidade Estadual de Goiás, 75132-903, Anápolis, Goiás, Brazil.

[c] Departmento de Física, Universidade Federal de Sergipe, 49107-230, São Cristóvão, Sergipe, Brazil.

[d] Grupo de Fotônica, Instituto de Física, Universidade Federal de Goiás, Goiânia, 74690-900, Goiás, Brazil.

*Corresponding author: lribeiro@ueg.br; luciana.ramos@ueg.br

Table of Contents

1. Synthesis and Spectroscopic Analysis... S2-23

2. UV-Vis Absorbance and Fluorescence Emission Spectra..…………………………........... S24-29

3. Optimized Cartesian Coordinates (Å) of Compounds... S30-S40

SM1 – Synthesis and spectroscopic analysis

All reagents and solvents were purchased from Aldrich and Merck and used without further purification. The reactions were conducted under heating and continuous stirring in a sealed Schlenk tube. Upon completion of the reaction, the resulting mixtures were stored at low temperature. Following the formation of a precipitate, the solid was isolated by vacuum filtration using ice-cold ethanol. The derivatives obtained were subsequently purified by recrystallization from ethanol. Reactions were monitored by thin-layer chromatography (TLC) on silica gel plates (Merck 60 GF254) using a hexane/ethyl acetate (1:1) eluent. Spots were visualized under ultraviolet (UV) light or developed with iodine (I_2). Melting points were determined using an MQAPF-301 Microchemistry apparatus and are reported without correction.

Infrared spectra were recorded on a Perkin Elmer Spectrum Frontier spectrometer using the KBr pellet method, with scans ranging from 4000 to 400 cm^{-1}. ^{1}H and ^{13}C{^{1}H} NMR spectra were acquired on a Bruker Avance III 500 (11.75T) spectrometer operating at 500 MHz for ^{1}H and 125 MHz for ^{13}C{^{1}H}. The instrument was equipped with ATB (automation triple resonance broadband) and SW (switchable) probes (5 mm internal diameter). Measurements were performed at room temperature with a pulse of 45 ºC for both hydrogen and carbon nuclei at the Institute of Chemistry, Samambaia Campus, Federal University of Goiás (IQ-UFG). Samples were dissolved in deuterated solvents (DMSO-d_6 or $CDCl_3$) with tetramethylsilane (TMS) as the internal standard.

3-(1*H*-Benzo[*d*]imidazol-2-yl)-2*H*-chromene-2-one (**19**): Orange solid with 60% yield (157 mg, 0.60 mmol), mp 233-234 °C (lit. 230-232 °C)[1]; IR (KBr) ν /cm^{-1} 3217, 2950, 2902, 2849, 1594, 1492, 1451, 1426, 1275, 1233, 1189, 1158, 1124, 1071, 1029, 883, 749, 746, 699 (**Figure S1**); ^{1}H NMR (500 MHz, DMSO-d_6) δ 10.09 (s, 1H, N-H), 8.94 (s, 1H, Bt-H*), 8.02-8.00 (d, 1H, J = 10.0 Hz, Bt-H*), 7.77 (t, 1H, J = 10.0 Hz, Ph-H), 7.55-7.46 (m, 3H, Bt-H*), 6.96 (t, 1H, J = 7.5 Hz, CHOH), 6.83-6.81 (d, 1H, J =10.0 Hz, CH_2), 6.65 (t, 1H, J = 7.5 Hz, CH_2) (**Figure S2**); ^{13}C{^{1}H} NMR (125 MHz, DMSO-d_6) δ 161.2, 160.2, 154.3, 141.8, 134.7, 130.8, 126.6, 125.8, 124.8, 123.8, 120.2, 118.9, 117.4, 116.8, 116.7 (**Figure S3**).

3-(benzo[*d*]thiazol-2-yl)-2*H*-chromene-2-one (**25**): Green solid with 24% yield (67 mg, 0.24 mmol), mp 218-219 ºC (lit. 215-217 °C)[2]; IR (KBr) ν /cm^{-1} 3050, 1718, 1610, 1566, 1480, 1319, 1187, 754 (**Figure S4**); ^{1}H NMR (500 MHz, $CDCl_3$) δ 9.10 (s, 1H, H-4), 8.10-8.12 (d, 1H, J = 10.0 Hz), 7.99-8.01 (d, 1H, J = 10.0 Hz), 7.75-7.76 (d, 1H, J = 5.0 Hz), 7.65-7.69 (t, 1H, J = 5.0 Hz), 7.54-7.57 (t,

1H, $J = 5.0$ Hz), 7.47-7.39 (m, 3H) (**Figure S5**);$^{13}C\{^1H\}$ NMR (125 MHz, $CDCl_3$) δ 159.5, 153.5, 141.2, 133.0, 129.1, 126.3, 125.1, 125.0, 122.6, 121.5, 116.7, 116.5 (**Figure S6**).

3-(2-methyl-2,3-dihydrobenzo[*d*]thiazol-2-yl)-2*H*-chromene-2-one (**26**): Yellow solid with 42% yield (124 mg, 0.42 mmol), mp 159-160 ºC (lit. 159-161 °C)[2]; IR (KBr) ν /cm^{-1} 3369, 3055, 2982, 2870, 1707, 1607, 1580, 1469, 1372, 1262, 1186, 747 (**Figure S7**); ^1H NMR (500 MHz, $CDCl_3$) δ 8.10 (s, 1H), 7.52 – 7.47 (m, 2H), 7.34 – 7.31 (m, 1H), 7.28 – 7.26 (m, 1H), 7.07 – 7.02 (m, 1H), 6.95 – 6.92 (m, 1H), 6.77 – 6.74 (m, 2H), 2.17 (s, 3H) (**Figure S8**); $^{13}C\{^1H\}$ NMR (125 MHz, $CDCl_3$) δ 138.6, 131.6, 128.5, 124.6, 121.9, 121.2, 116.3, 111.3, 28.2 (**Figure S9**) and ^1H-^{13}C HMBC **(Figure S10).**

3-(1*H*-benzo[*d*]imidazol-2-carbonyl)-2*H*-chromene-2-one (**27**): Yellow solid with 48% yield (139 mg, 0.48 mmol), mp 213-214 ºC; IR (KBr) ν /cm^{-1} 3416, 3068, 1723, 1613, 1568, 1489, 1199, 1100 (**Figure S11**); ^1H NMR (500 MHz, $CDCl_3$) δ 9.83 (s, 1H), 8.88 (s, 1H), 8.19-8.15 (m, 2H), 7.84-7.80 (m, 2H), 7.74-7.75 (d, 1H, $J = 5.0$ Hz), 7.64-7.67 (t, 1H, $J = 5.0$ Hz), 7.45-7.47 (d, 1H, $J = 10.0$ Hz), 7.37-7.40 (t, 1H, $J = 5.0$ Hz) (**Figure S12**); $^{13}C\{^1H\}$ NMR (125 MHz, $CDCl_3$) δ 160.2, 154.4, 147.4, 145.5, 144.6, 142.2, 132.9, 130.4, 130.3, 129.4, 129.2, 124.9, 124.1, 119.4, 116.7 (**Figure S13**) and ^1H-^{13}C HMBC **(Figure S14).**

3-(1*H*-benzo[*d*]imidazol-2-yl)-7-hydroxy-2*H*-chromen-2-one (**28**): Yellow solid with 50% yield (139 mg, 0.5 mmol), mp > 290 ºC (lit. 294 °C)[3]; IR (KBr) ν /cm^{-1} 3410, 3334, 3072, 1716, 1599, 1518, 1419, 1254, 1132 (**Figure S15**); ^1H NMR (500 MHz, DMSO-d_6) δ 12.40 (s, 1H), 9.04 (s, 1H), 7.83 (d, $J = 8.6$ Hz, 1H), 7.67 – 7.62 (m, 2H), 7.20 (dd, $J = 6.0, 3.1$ Hz, 3H), 6.90 (dd, $J = 8.5, 2.2$ Hz, 1H), 6.85 (d, $J = 2.0$ Hz, 1H) (**Figure S16**); $^{13}C\{^1H\}$ NMR (125 MHz, DMSO-d_6) δ 162.4, 159.4, 155.2, 146.1, 142.7, 142.6, 134.4, 130.9, 122.0, 121.7, 117.9, 113.9, 112.3, 111.5, 111.3, 101.7 (**Figure S17**).

7-hydroxy-3-(6-methyl-1*H*-benzo[*d*]imidazol-2-yl)-2*H*-chromen-2-one (**29**): Yellow solid with 39% yield (113 mg, 0.39 mmol), mp >290 ºC. IR (KBr) ν /cm^{-1} 3437, 3344, 3080, 2911, 2852, 1702, 1613, 1572, 1504, 1265, 1132 (**Figure S18**); ^1H NMR (500 MHz, DMSO-d_6) δ 12.26 (d, $J = 13.2$ Hz, 1H), 8.99 (s, 1H), 7.82 (d, $J = 8.3$ Hz, 1H), 7.51 (t, $J = 8.4$ Hz, 1H), 7.43 (d, J = 7.5 Hz, 1H), 7.02 (d, J = 8.2 Hz, 1H), 6.89 (dd, $J = 8.5, 2.2$ Hz, 1H), 6.84 (d, $J = 2.0$ Hz, 1H), 2.42 (s, 3H) (**Figure S19**); $^{13}C\{^1H\}$ NMR (125 MHz, DMSO-d_6) δ 161.9, 159.1, 155.1, 142.2, 142.1, 134.5, 130.7, 123.4, 117.4, 117.3, 113.6, 111.7, 111.4, 111.2, 101.5, 20.7 (**Figure S20**).

3-(benzo[*d*]thiazol-2-yl)-7-hydroxy-2*H*-chromen-2-one (**<u>30</u>**): Yellow solid with 27% yield (79.7 mg, 0.27 mmol), mp >290 ºC (lit. 302-304 °C)[4]; IR (KBr) ν /cm^{-1} 3437, 3065, 1723, 1599, 1565, 1448, 1242, 1199, 766 (**Figure S21**); ^{1}H NMR (500 MHz, DMSO-d_6) δ 9.15 (s, 1H), 8.14-8.16 (d, 1H, J = 5.0 Hz), 8.04-8.05 (d, 1H, J = 5.0 Hz), 7.90-7.92 (d, 1H, J = 5.0 Hz), 7.54-7.57 (t, 1H, J = 5.0 Hz), 7.44-7.47 (t, 1H, J = 5.0 Hz), 6.91-6.3 (d, 1H, J = 10.0 Hz), 6.85 (s, 1H) (**Figure S22**); ^{13}C{^{1}H} NMR (125 MHz, DMSO-d_6) δ 163.7, 160.6, 160.0, 155.9, 152.2, 142.8, 135.8, 132.1, 126.7, 125.2, 122.3, 114.7, 111.6, 102.2 (**Figure S23**).

3-(1*H*-benzo[*d*]imidazol-2-yl)-6-bromo-2*H*-chromen-2-one (**<u>31</u>**): Yellow solid with 45% yield (153.5 mg, 0.45 mmol), mp 240-241 ºC (lit. 276-277 °C)[5]; IR (KBr) ν /cm^{-1} 3355, 3043, 1705, 1663, 1593, 1552, 1240 (**Figure S24**); ^{1}H NMR (500 MHz, DMSO-d_6) δ 10.04 (s, 1H), 8.90 (s, 1H), 8.29 (d, J = 2.4 Hz, 1H), 7.91 (dd, J = 8.8, 2.4 Hz, 1H), 7.56 – 7.48 (m, 2H), 7.00 – 6.94 (m, 1H), 6.82 (dd, J = 8.0, 1.1 Hz, 1H), 6.66 – 6.63 (m, 1H) (**Figure S25**); ^{13}C{^{1}H} NMR (125 MHz, DMSO-d_6) δ 160.1, 159.5, 152.9, 145.9, 141.4, 136.3, 132.1, 126.2, 124.3, 120.3, 118.5, 116.8, 116.2 (**Figure S26**).

3-(benzo[*d*]thiazol-2-yl)-6-bromo-2*H*-chromen-2-one (**<u>32</u>**): Yellow solid with 14% yield (50 mg, 0.14 mmol), mp 268-269 ºC (lit. 260-261 °C)[6]; IR (KBr) ν /cm^{-1} 3071, 1753, 1705, 1615, 1559, 1240 (**Figure S27**); ^{1}H NMR (500 MHz, DMSO-d_6) δ 11.71 (s, 1H), 8.39 (d, J = 2.6 Hz, 1H), 8.15 (d, J = 7.7 Hz, 1H), 8.08 (d, J = 8.1 Hz, 1H), 7.56 (ddd, J = 8.2, 3.8, 1.6 Hz, 2H), 7.48 – 7.45 (m, 1H), 7.07 (d, J = 8.8 Hz, 1H).(**Figure S28**); ^{13}C{^{1}H} NMR (125 MHz, DMSO-d_6) δ 155.3, 151.3, 135.1, 134.5, 130.2, 126.5, 125.2, 122.4, 122.0, 121.0, 119.2, 110.9 (**Figure S29**).

3-(1*H*-benzo[*d*]imidazol-2-yl)-7-(diethylamino)-2*H*-chromene-2-one (**<u>33</u>**): Yellow solid with 45% yield (149 mg, 0.45 mmol), mp 223-224 °C (lit. 218-220 °C)[7]; IR (KBr) ν /cm^{-1} 3431, 3022, 2981, 2925, 1718, 1635, 1593, 1524, 1268 (**Figure S30)**. ^{1}H NMR (500 MHz, DMSO-d_6) 8,92 (s, 1H), 8,55 (s, 1H), 7,72 (d, J = 8,9 Hz, 1H), 7,64 – 7,58 (m, 1H), 7,51 (d, J = 9,0 Hz, 1H), 7,17 – 7,15 (m, 1H), 6,84 (d, J = 9,0 Hz, 1H), 6,67 – 6,61 (m, 1H), 3,49 (q, J = 6,9 Hz, 4H), 1,18-1,12 (m, 6H). (**Figure S31**); ^{13}C{^{1}H} NMR (125 MHz, DMSO-d_6) δ 159.7, 158.5, 157.5, 155.0, 153.3, 152.0, 131.4, 125.9, 116.2, 110.4, 109.8, 107.0, 96.5, 44.5, 44.2, 12.3 (**Figure S32**).

3-(benzo[*d*]thiazol-2-yl)-7-(diethylamino)-2*H*-chromene-2-one (**<u>34</u>**): Yellow solid with 26% yield (119 mg, 0.34 mmol), mp 203-207 °C (lit. 198-202 °C)[8]; IR (KBr) ν /cm^{-1} 3015, 2981, 2939, 1718,

1635, 1587, 1517, 1268 (**Figure S33**). ^{1}H NMR (500 MHz, DMSO-d_6) δ 8,55 (s, 1H), 7,72 (d, J = 8,9 Hz, 1H), 7,64 – 7,59 (m, 1H), 7,52 (d, J = 9,0 Hz, 1H), 7,17 – 7,15 (m, 1H), 6,84 (d, J = 9,0 Hz, 1H), 6,66 – 6,61 (m, 1H), 3,50(q, J = 6,9 Hz, 4H), 1,16-1,12 (m, 6H), (**Figure S34**); ^{13}C{^{1}H} NMR (125 MHz, DMSO-d_6) δ 157.9, 155.5, 153.7, 152.4, 131.8, 116.7, 110.8, 107.4, 96.9, 44.9, 12.8 (**Figure S35**).

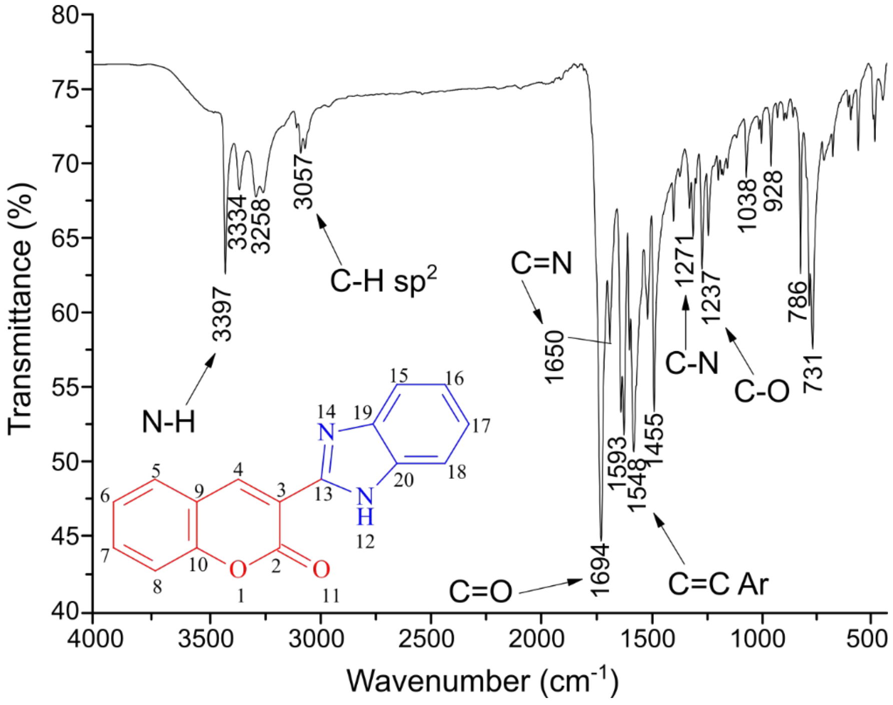

Figure S1. FTIR (KBr) spectrum of compound **19**.

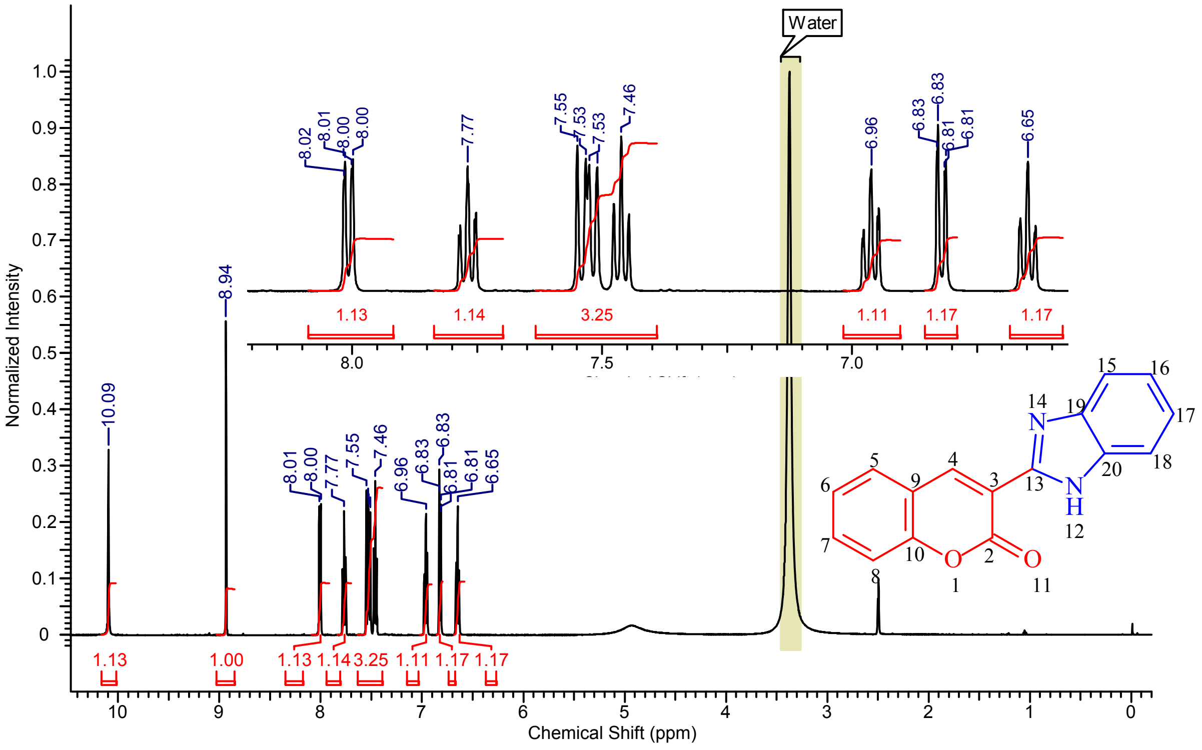

Figure S2. ^{1}H NMR spectrum (500 MHz, DMSO-d_6) of compound **19**.

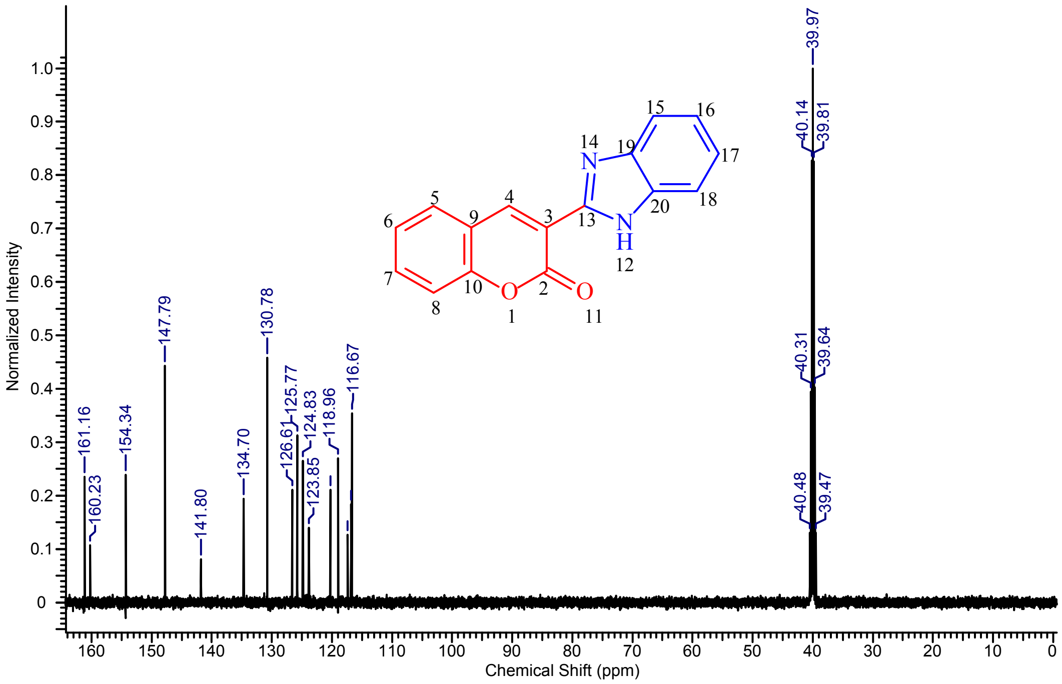

Figure S3. $^{13}C\{^{1}H\}$ NMR spectrum (125 MHz, DMSO-d_6) of compound **19**.

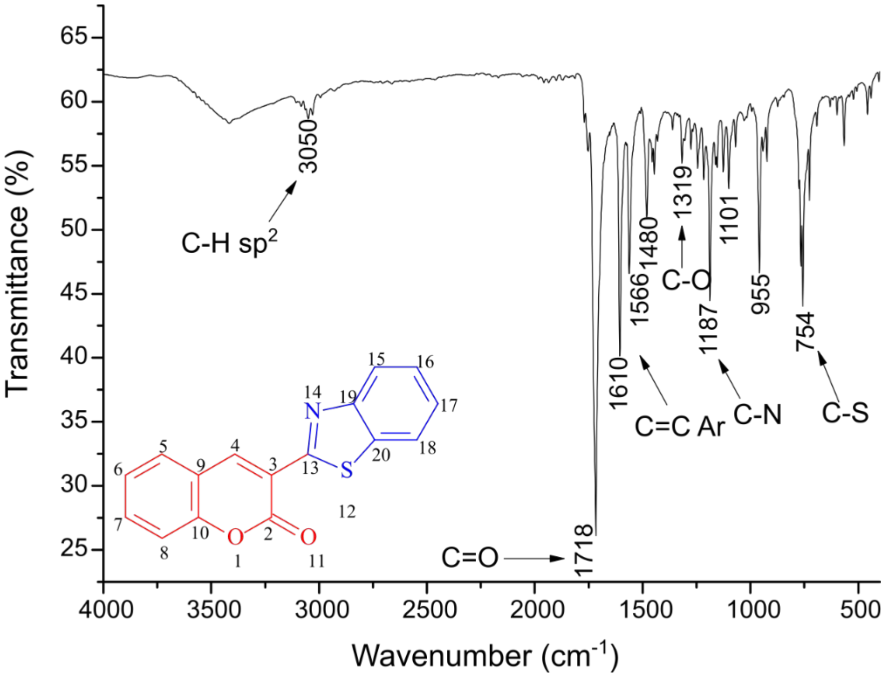

Figure S4. FTIR (KBr) spectrum of compound **25**.

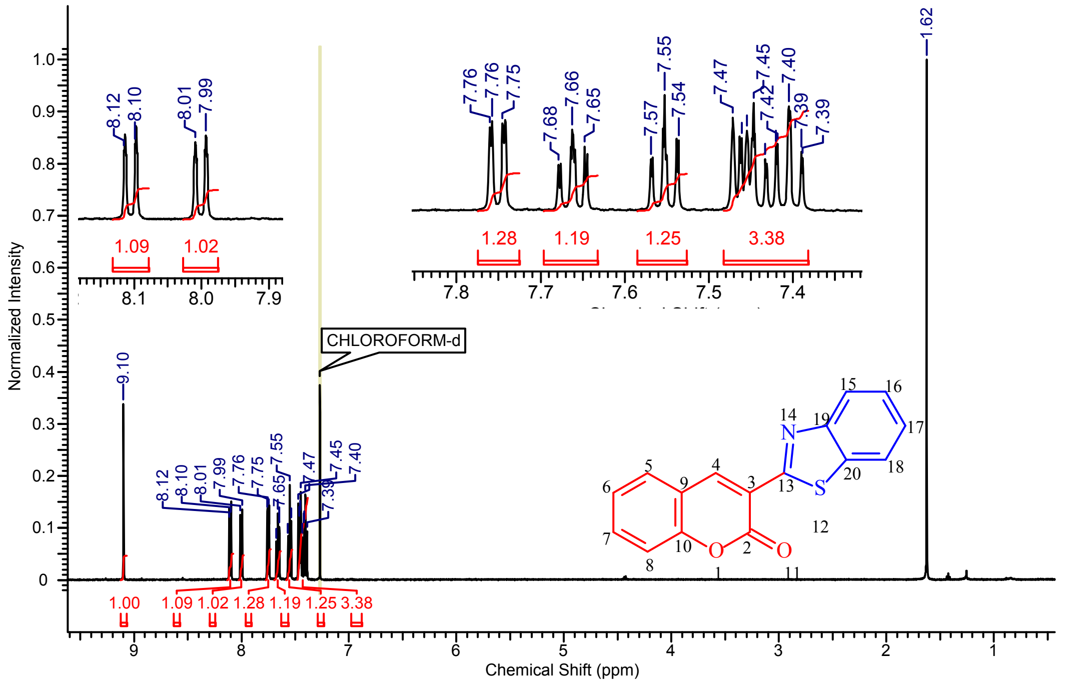

Figure S5. ^{1}H NMR spectrum (500 MHz, $CDCl_3$) of compound **25**.

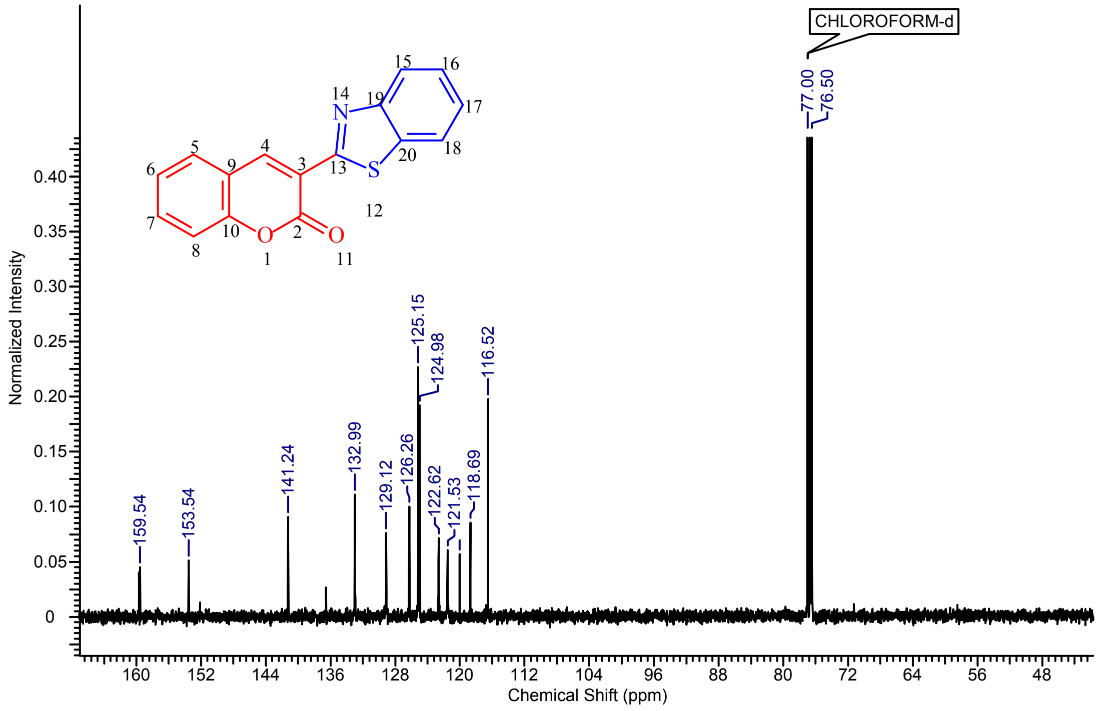

Figure S6. $^{13}C\{^{1}H\}$ NMR spectrum (125 MHz, $CDCl_3$) of compound **25**.

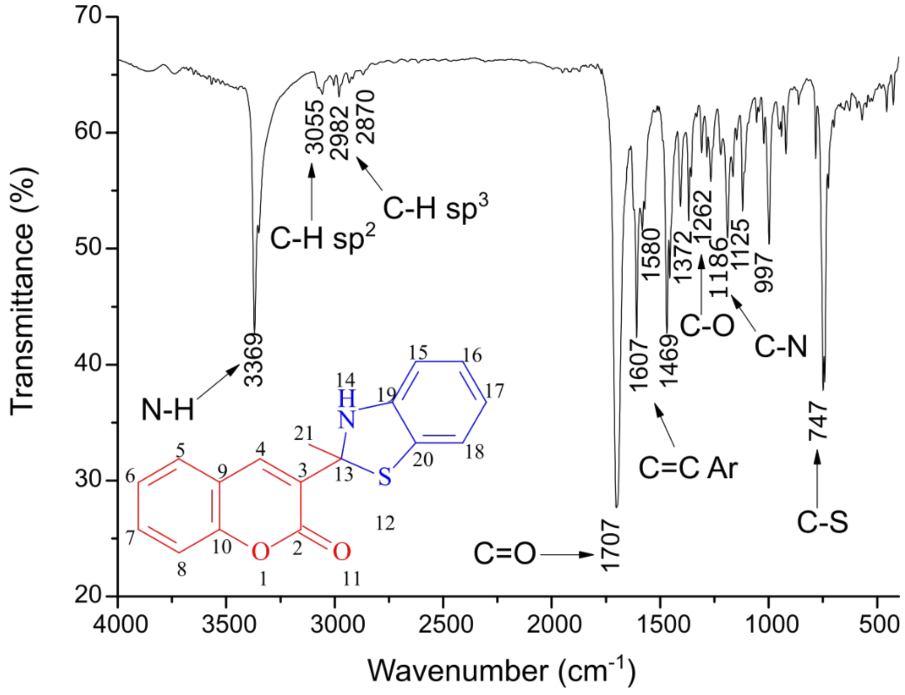

Figure S7. FTIR (KBr) spectrum of compound **26**.

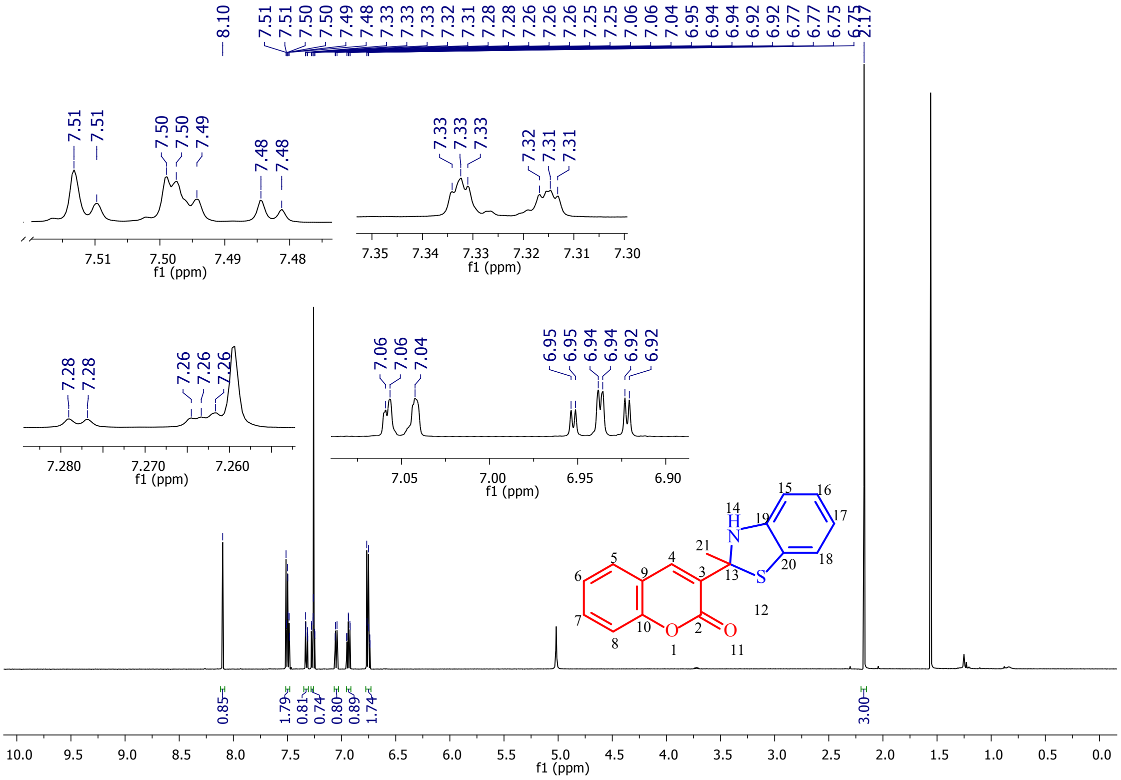

Figure S8. ^{1}H NMR spectrum (500 MHz, $CDCl_3$) of compound **26**.

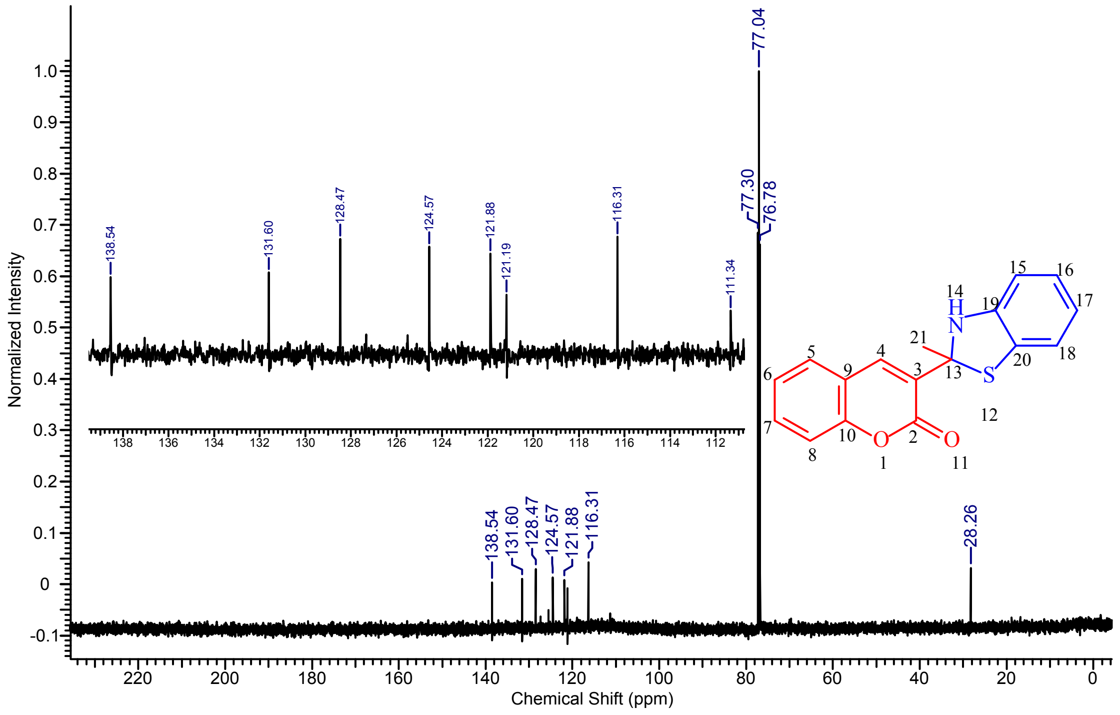

Figure S9. $^{13}C\{^{1}H\}$ NMR spectrum (125 MHz, $CDCl_3$) of compound **26**.

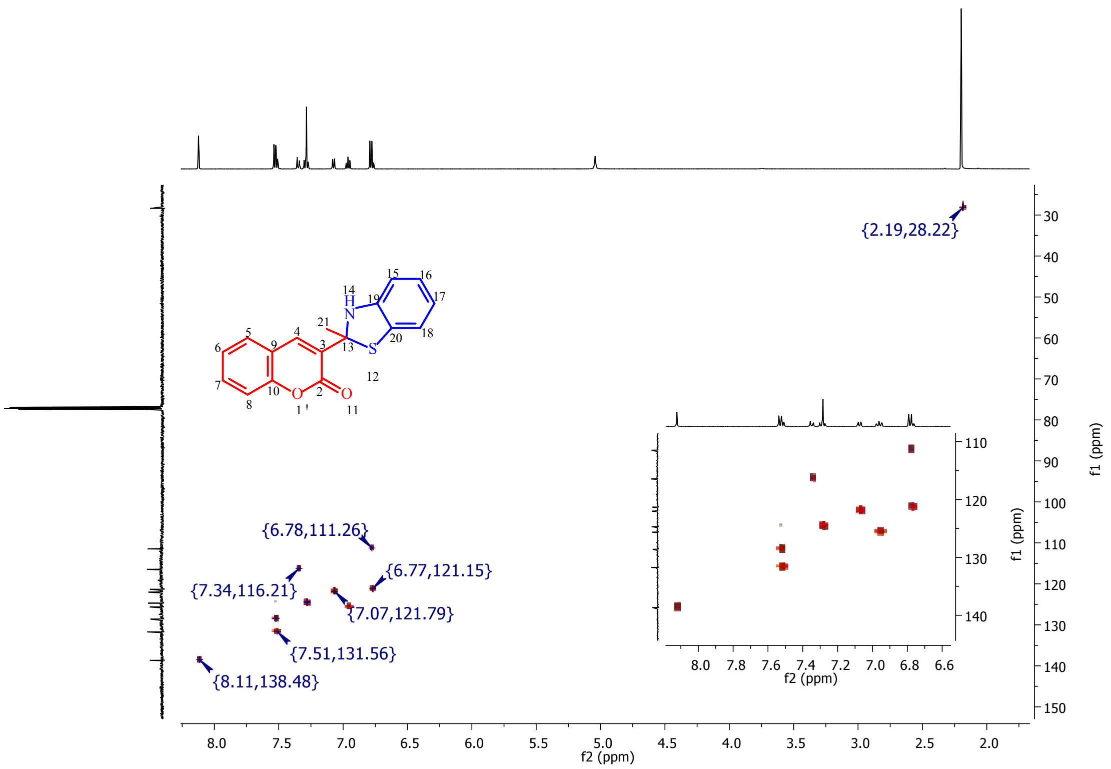

Figure S10. ^{1}H-^{13}C HMBC spectra of compound **26**.

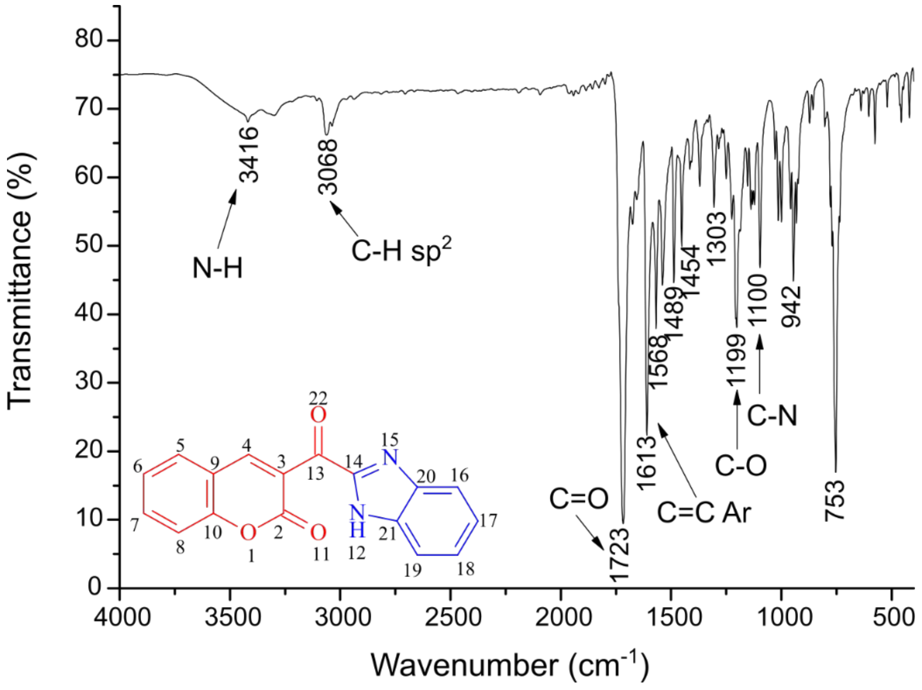

Figure S11. FTIR (KBr) spectrum of compound **27**.

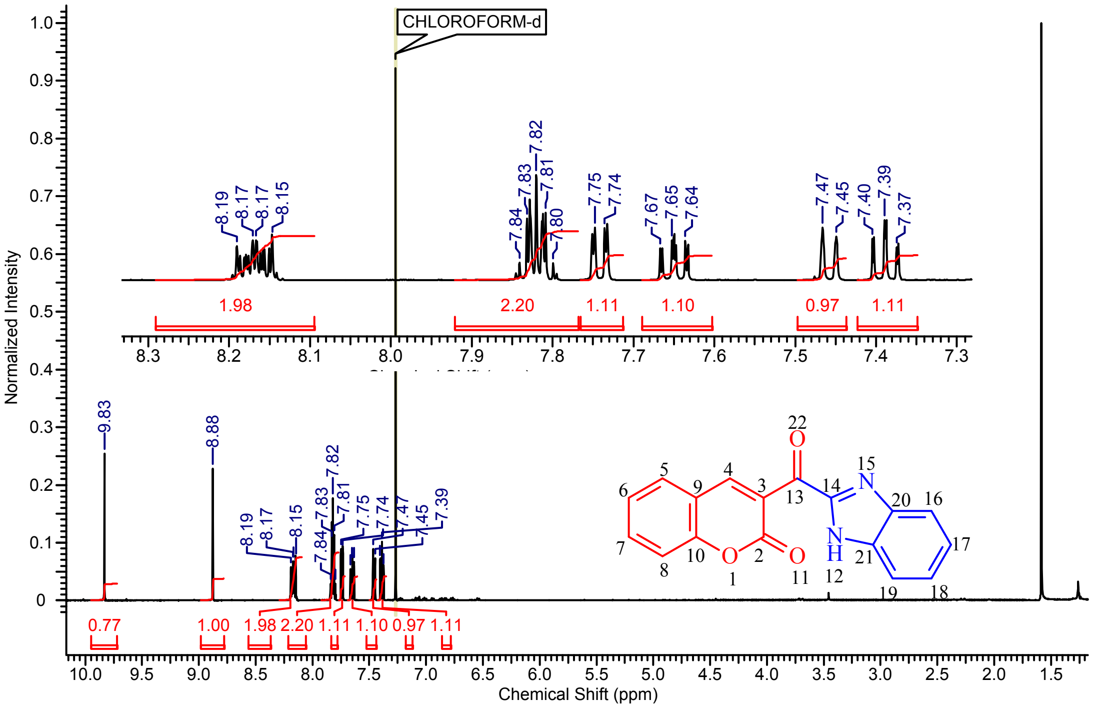

Figure S12. ^{1}H NMR spectrum (500 MHz, $CDCl_3$) of compound **27**.

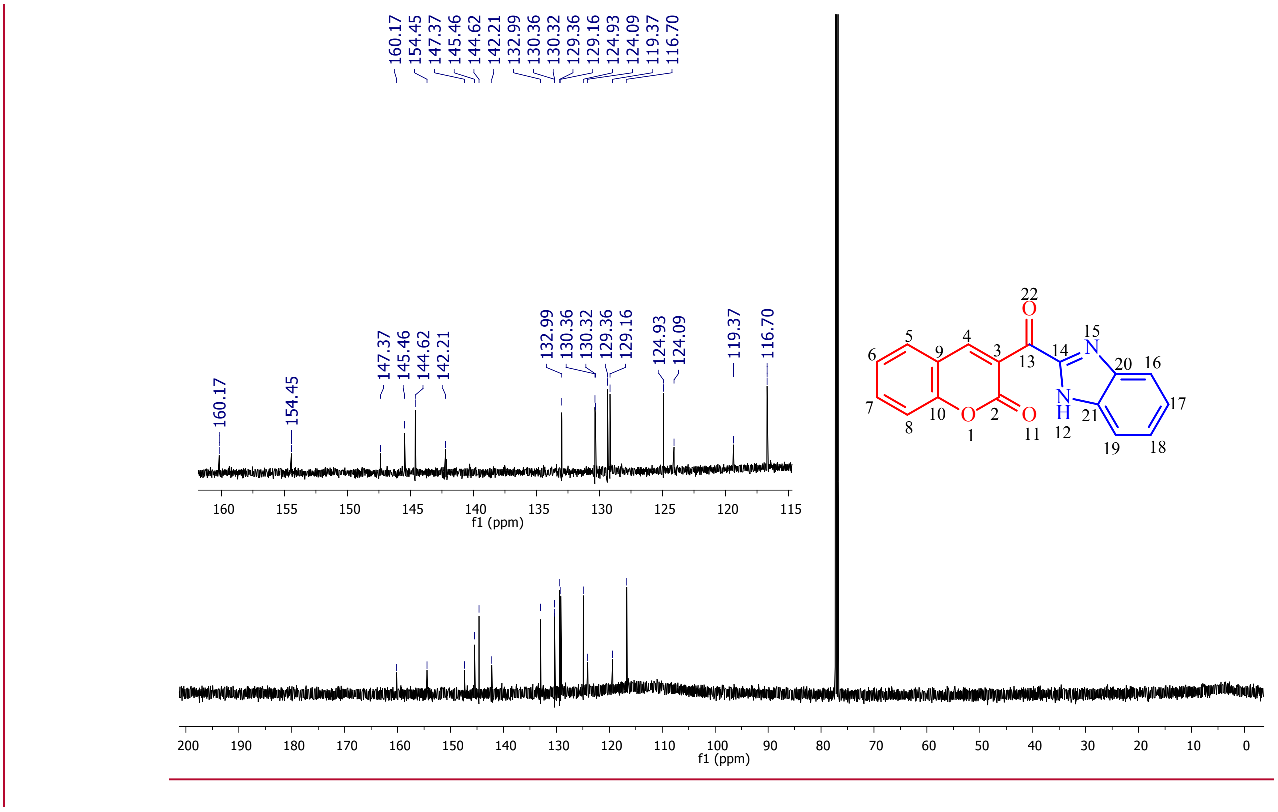

Figure S13. $^{13}C\{^{1}H\}$ NMR spectrum (125 MHz, $CDCl_3$) of compound **<u>27</u>**.

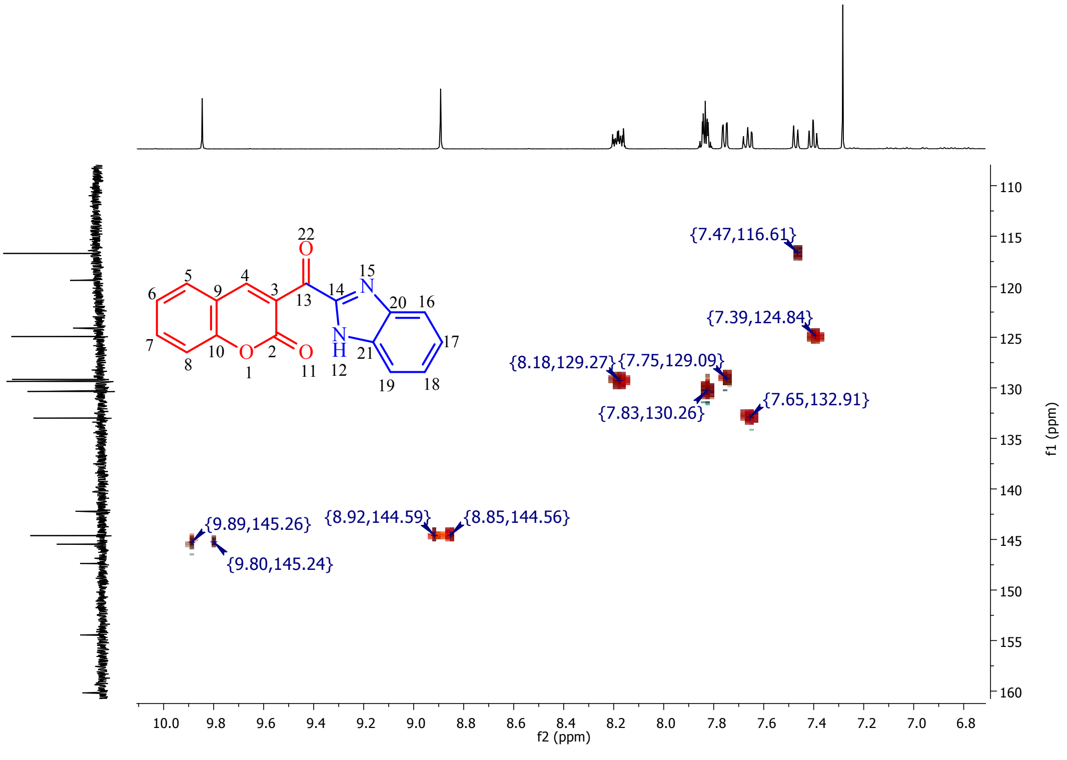

Figure S14. ^{1}H-^{13}C HMBC spectra of compound **<u>27</u>.**

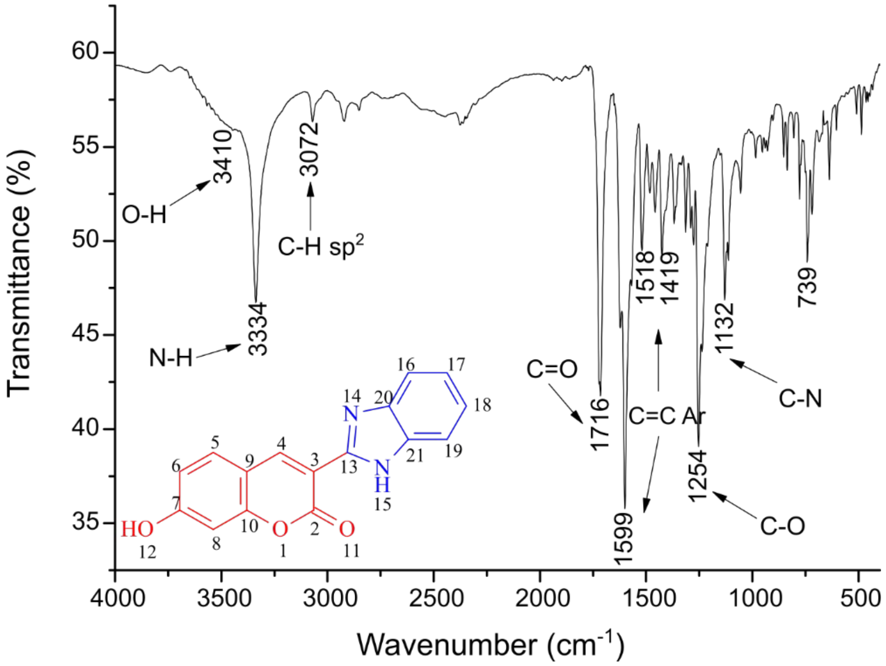

Figure S15. FTIR (KBr) spectrum of compound **28**.

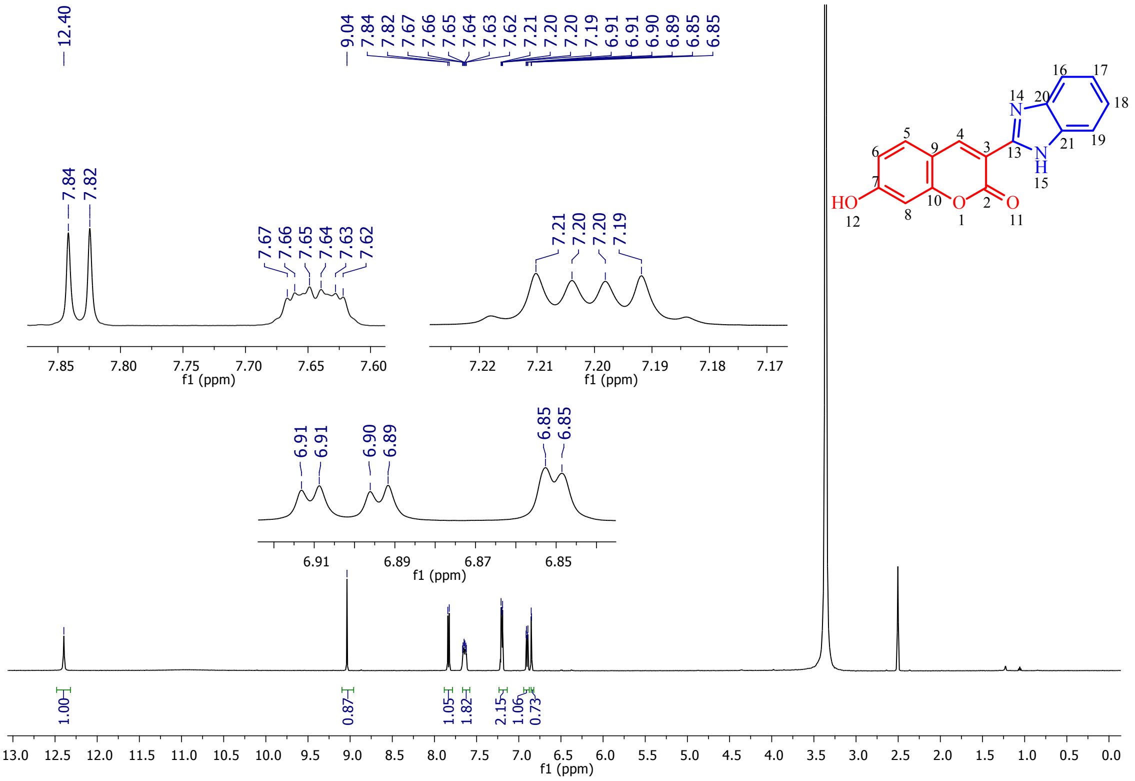

Figure S16. ^{1}H NMR spectrum (500 MHz, DMSO-*d*6) of compound **28**.

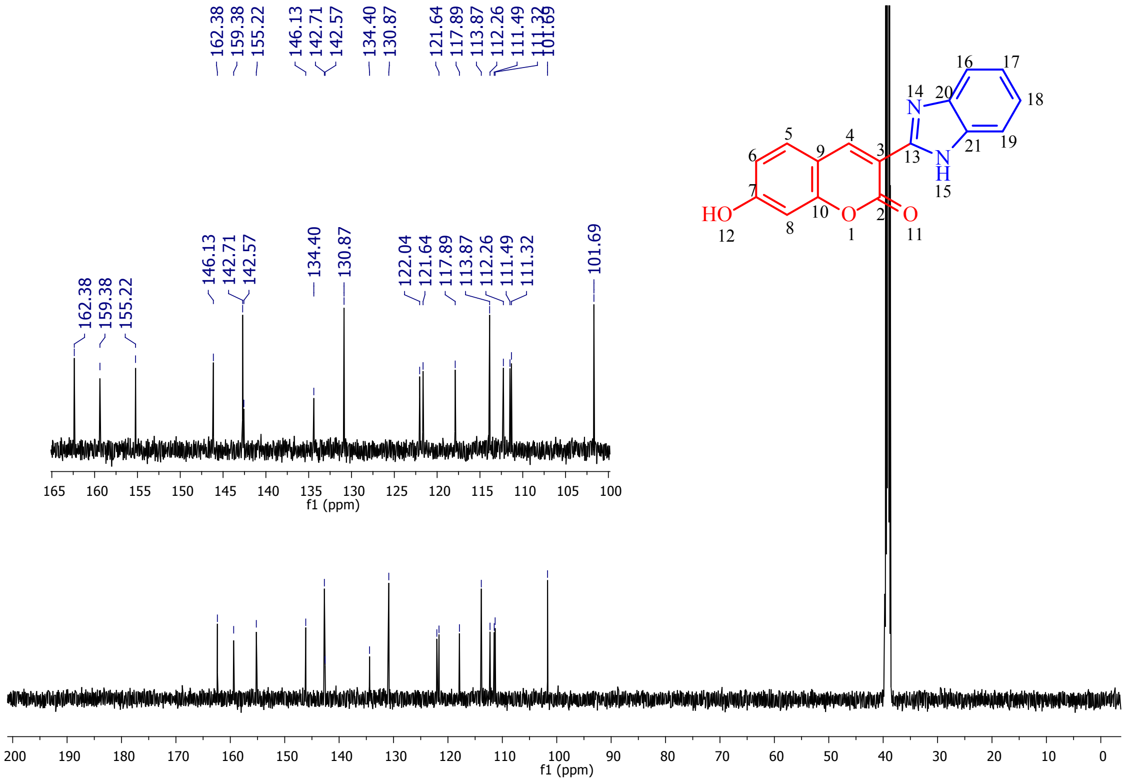

Figure S17. $^{13}C\{^1H\}$ NMR spectrum (125 MHz, DMSO-*d6*) of compound **28**.

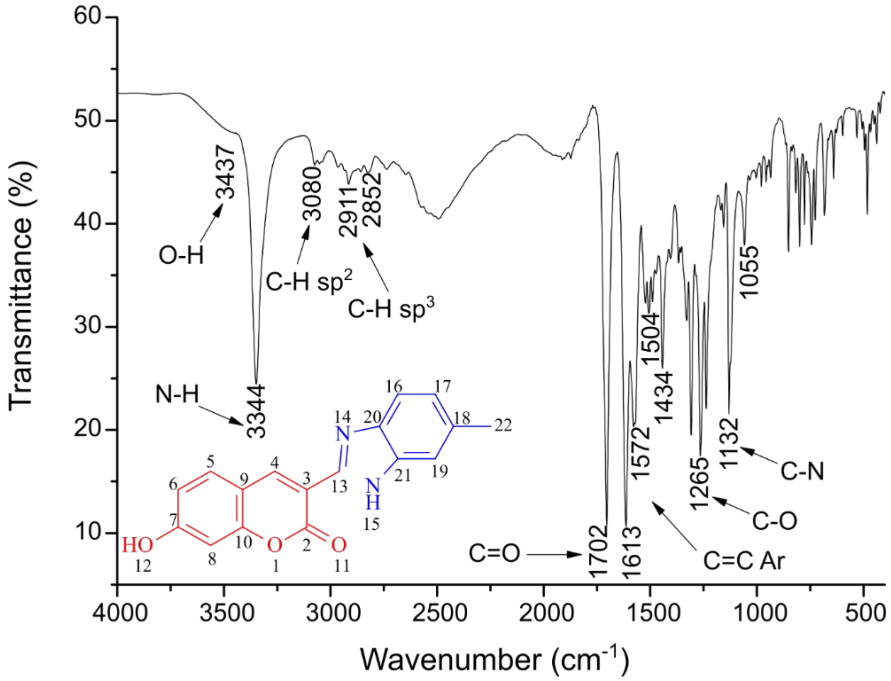

Figure S18. FTIR (KBr) spectrum of compound **29**.

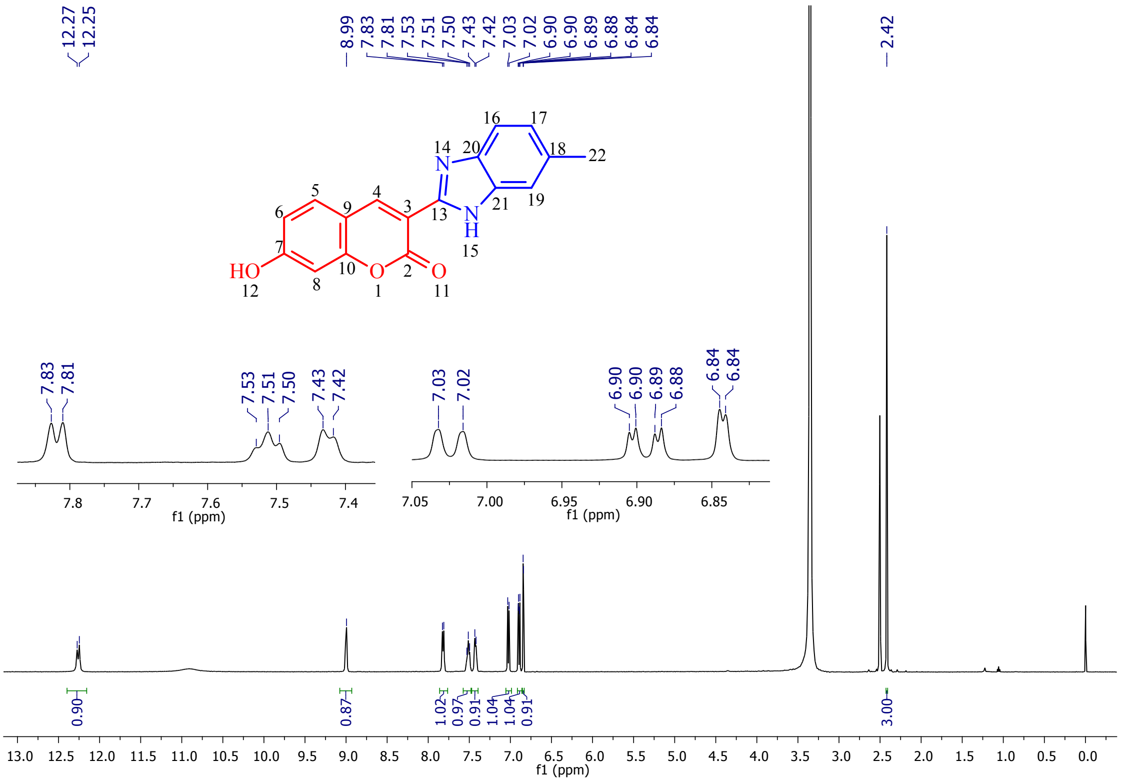

Figure S19. ^{1}H NMR spectrum (500 MHz, DMSO-*d*6) of compound **29**.

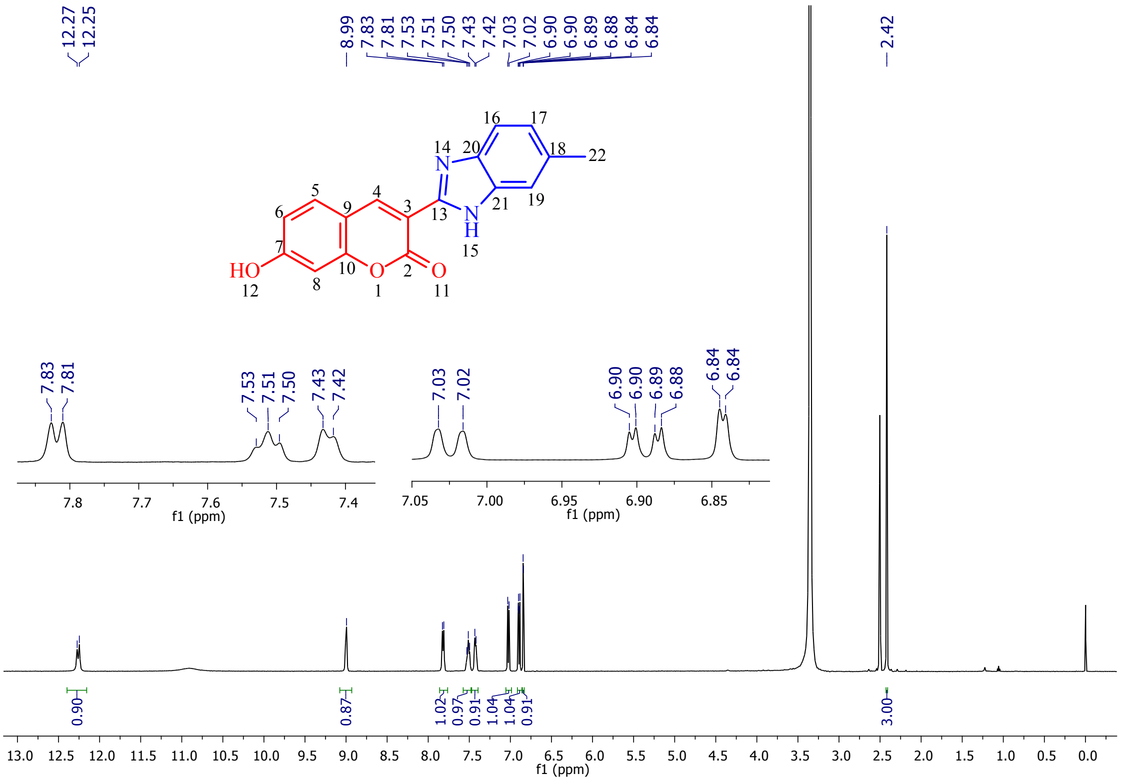

Figure S20. $^{13}C\{^{1}H\}$ NMR spectrum (125 MHz, DMSO-*d*6) of compound **29**.

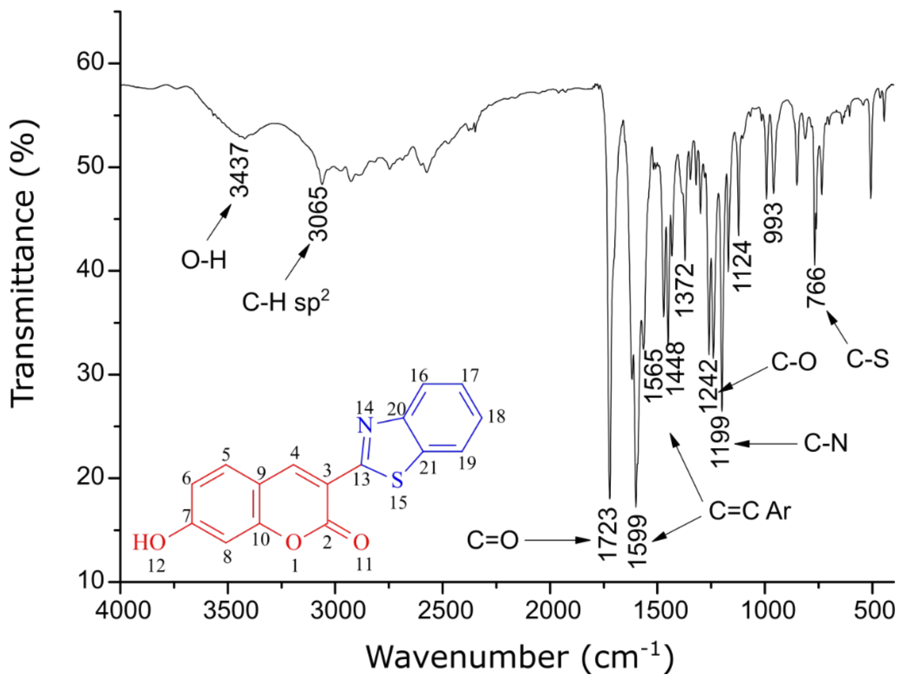

Figure S21. FTIR (KBr) spectrum of compound **30**.

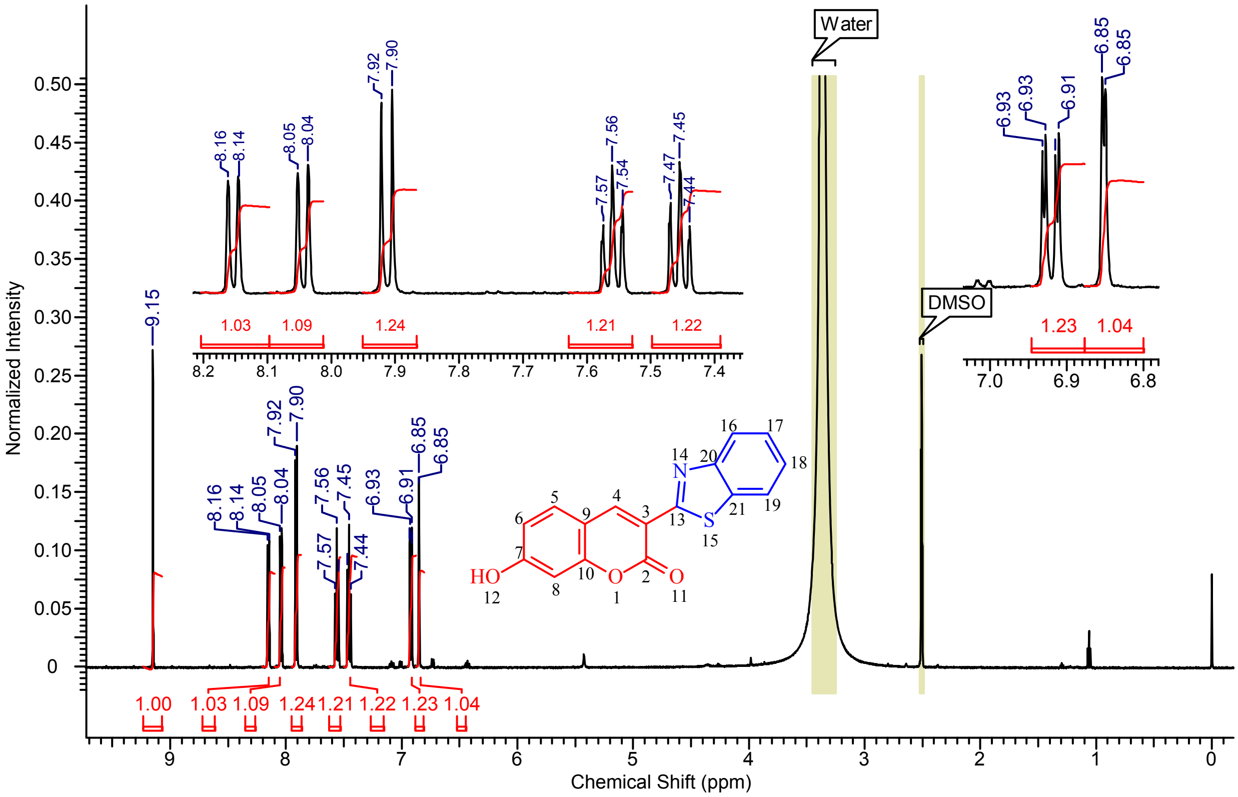

Figure S22. ^{1}H NMR spectrum (500 MHz, DMSO-*d6*) of compound **30**.

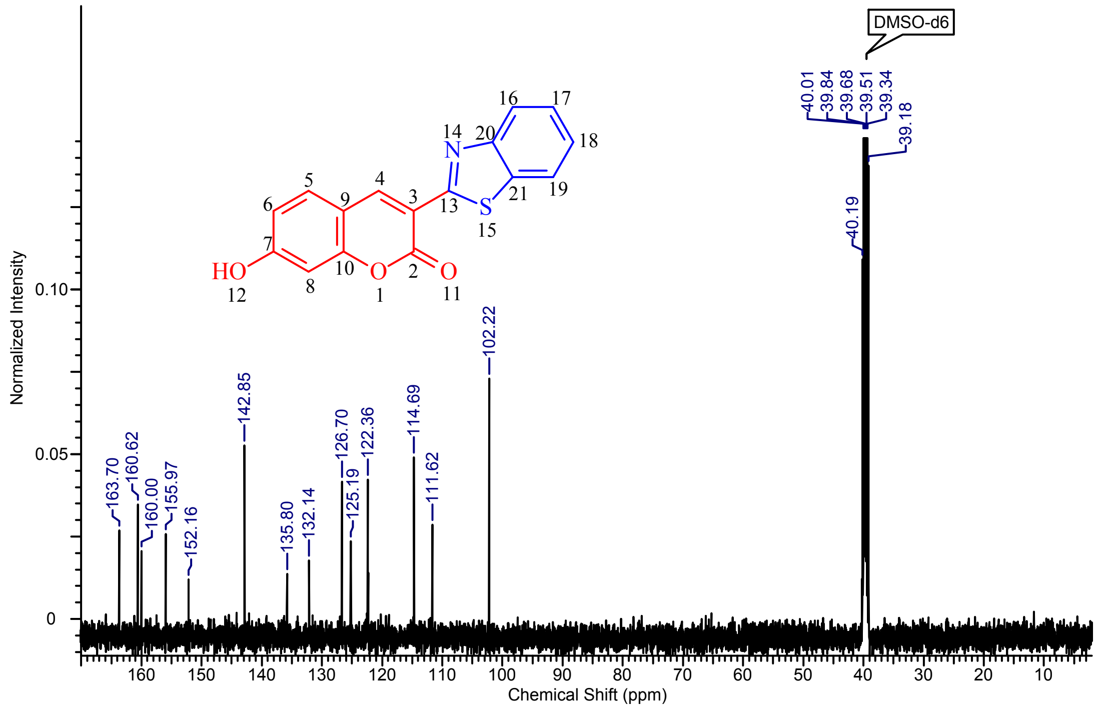

Figure S23. $^{13}C\{^{1}H\}$ NMR spectrum (125 MHz, DMSO-*d*6) of compound **30**.

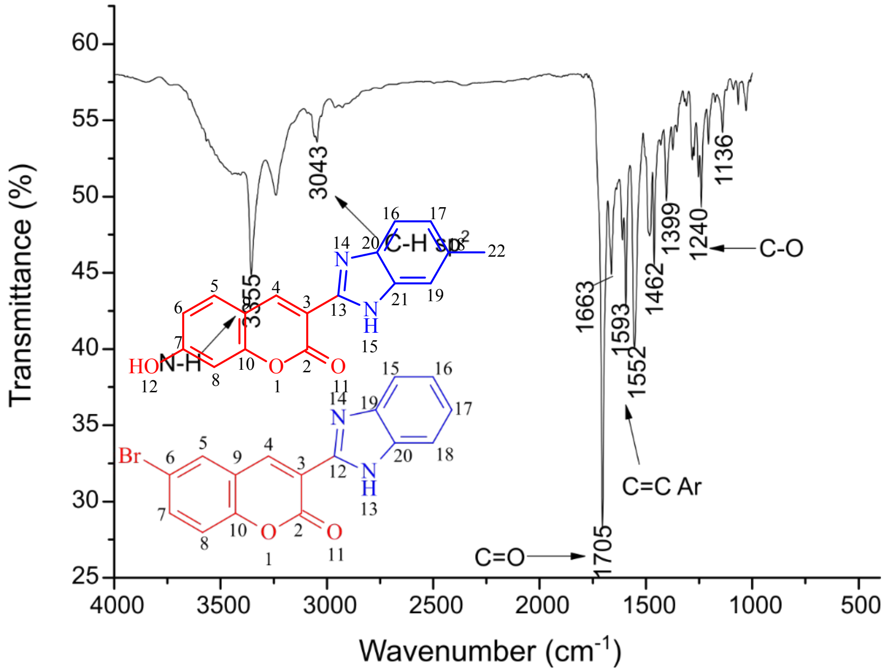

Figure S24. FTIR (KBr) spectrum of compound **31**.

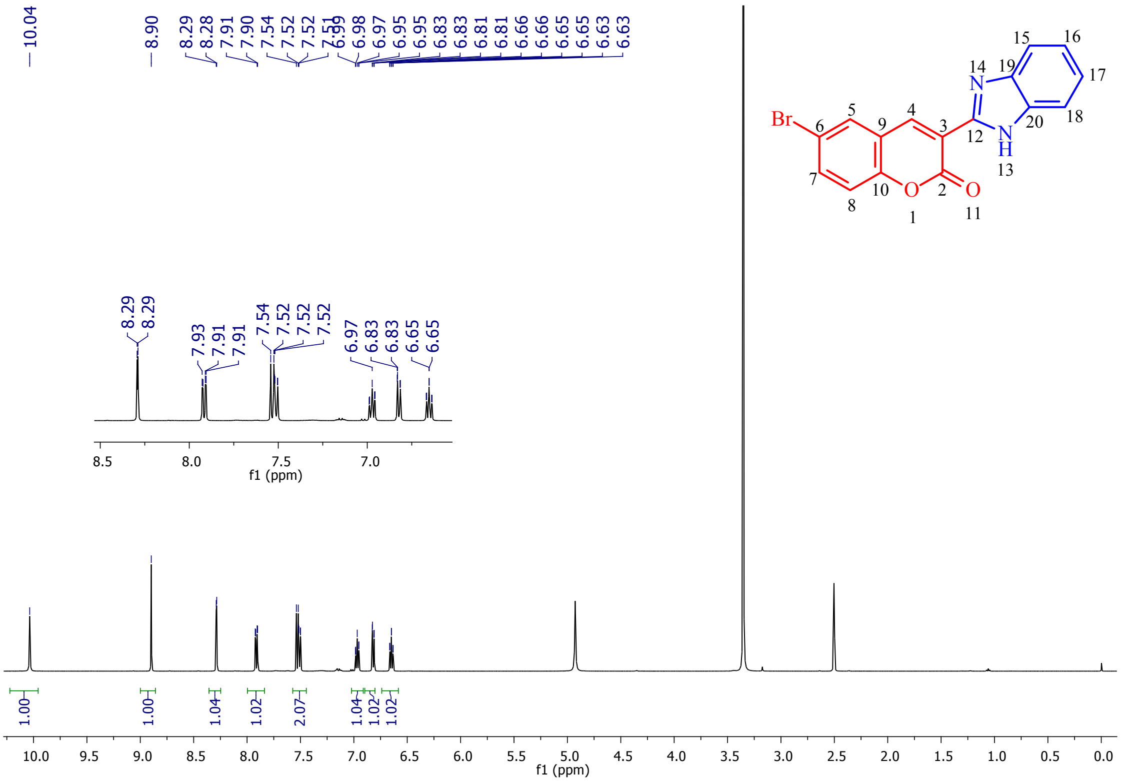

Figure S25. ^{1}H NMR spectrum (500 MHz, DMSO-*d*6) of compound **31**.

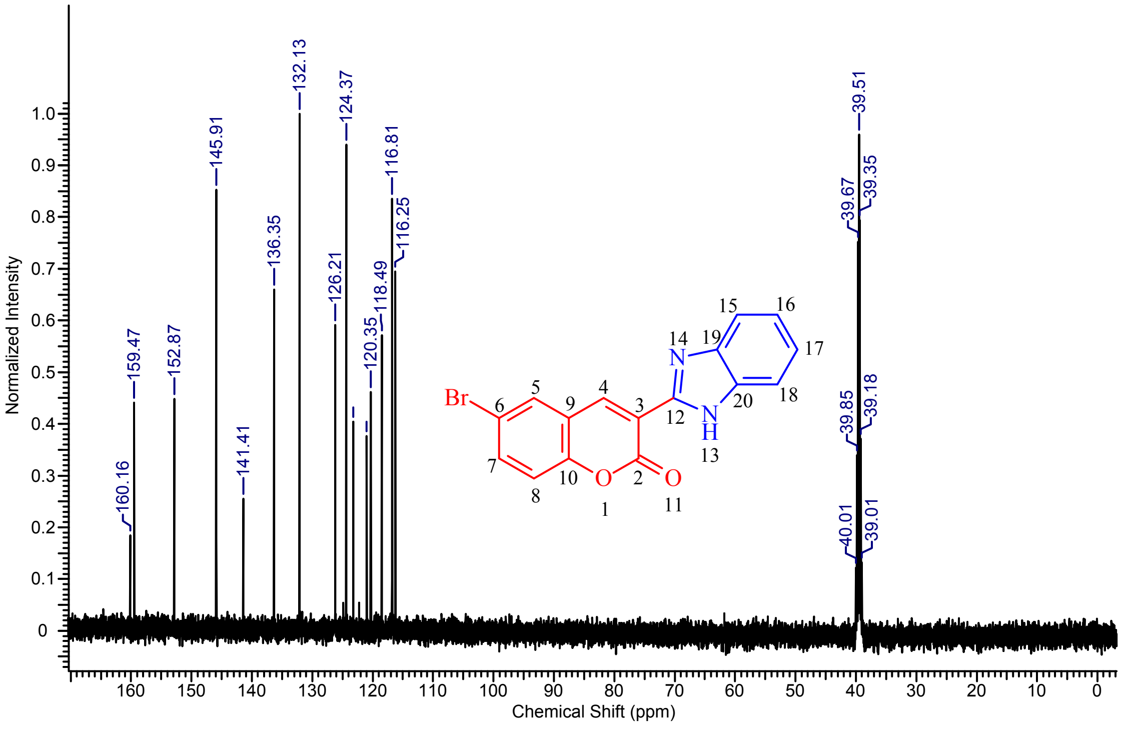

Figure S26. $^{13}C\{^{1}H\}$ NMR spectrum (125 MHz, DMSO-*d*6) of compound **31**.

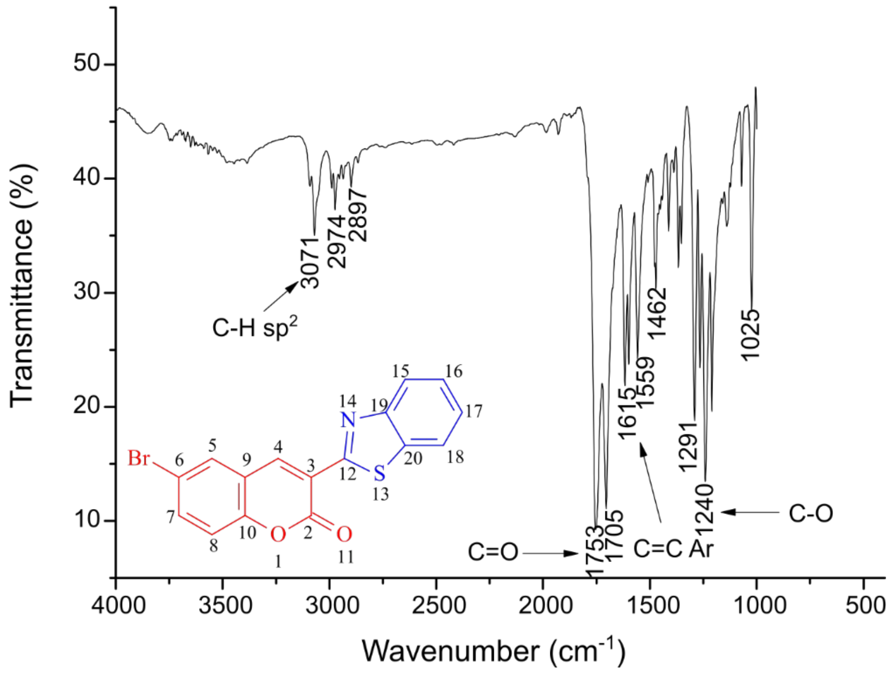

Figure S27. FTIR (KBr) spectrum of compound **32**.

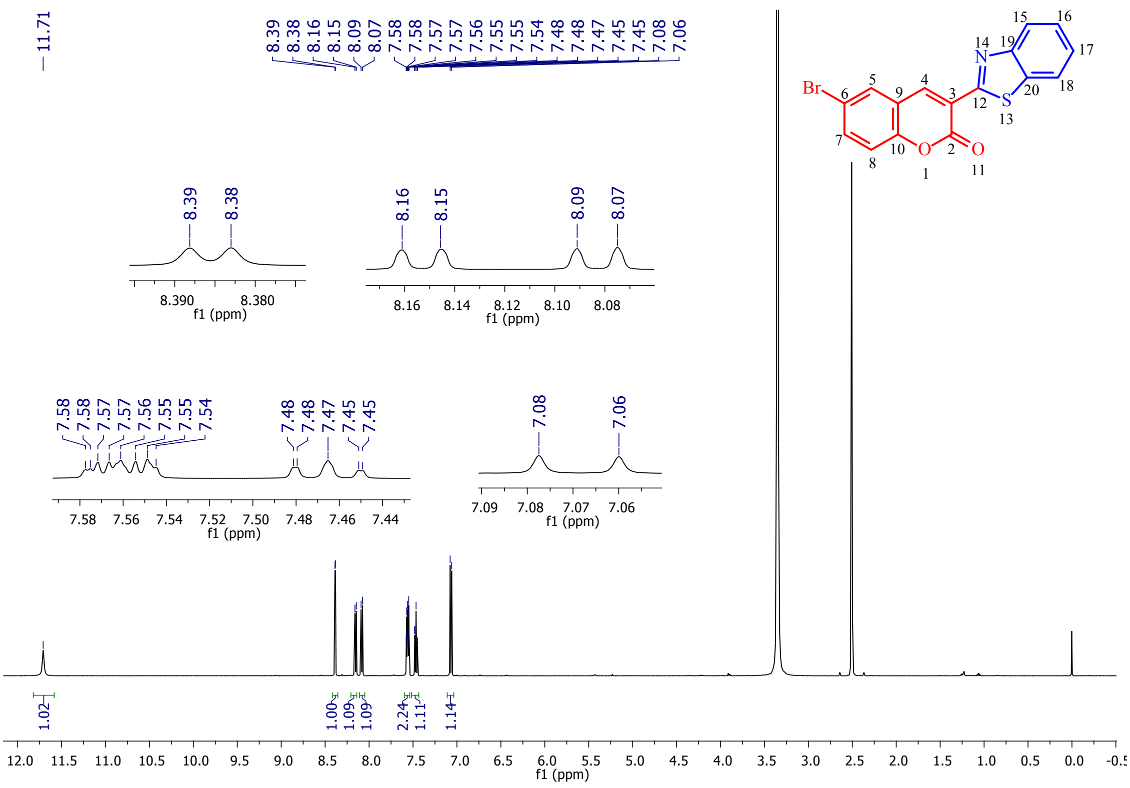

Figure S28. ^{1}H NMR spectrum (500 MHz, DMSO-*d6*) of compound **32**.

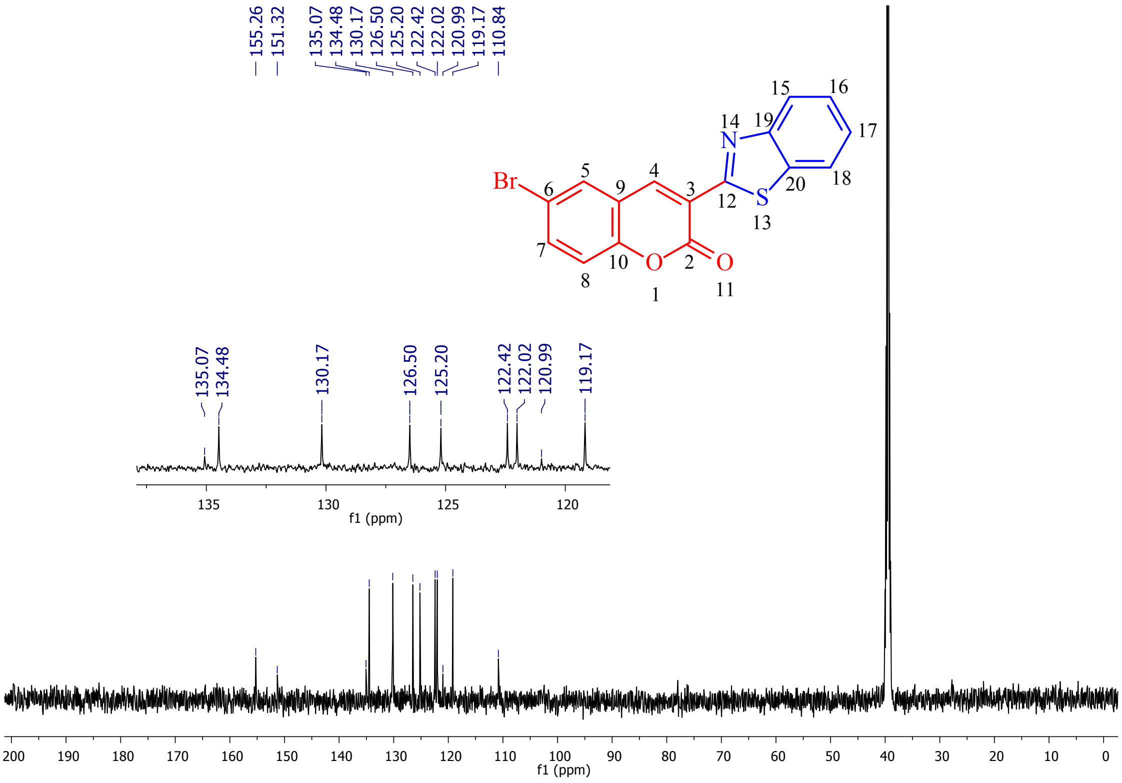

Figure S29. $^{13}C\{^{1}H\}$ NMR spectrum (125 MHz, DMSO-*d6*) of compound **32**.

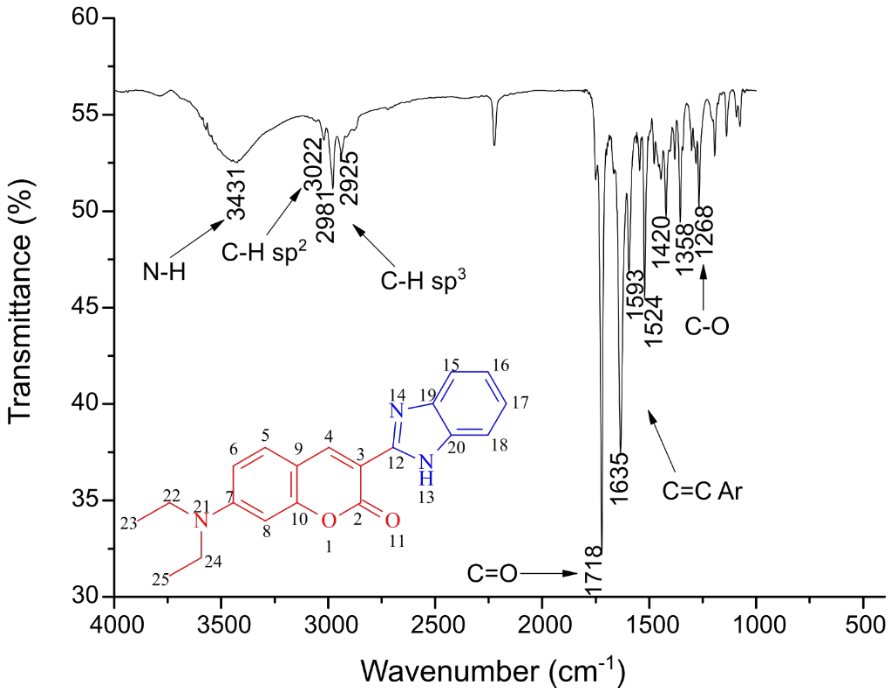

Figure S30. FTIR (KBr) spectrum of compound **33**.

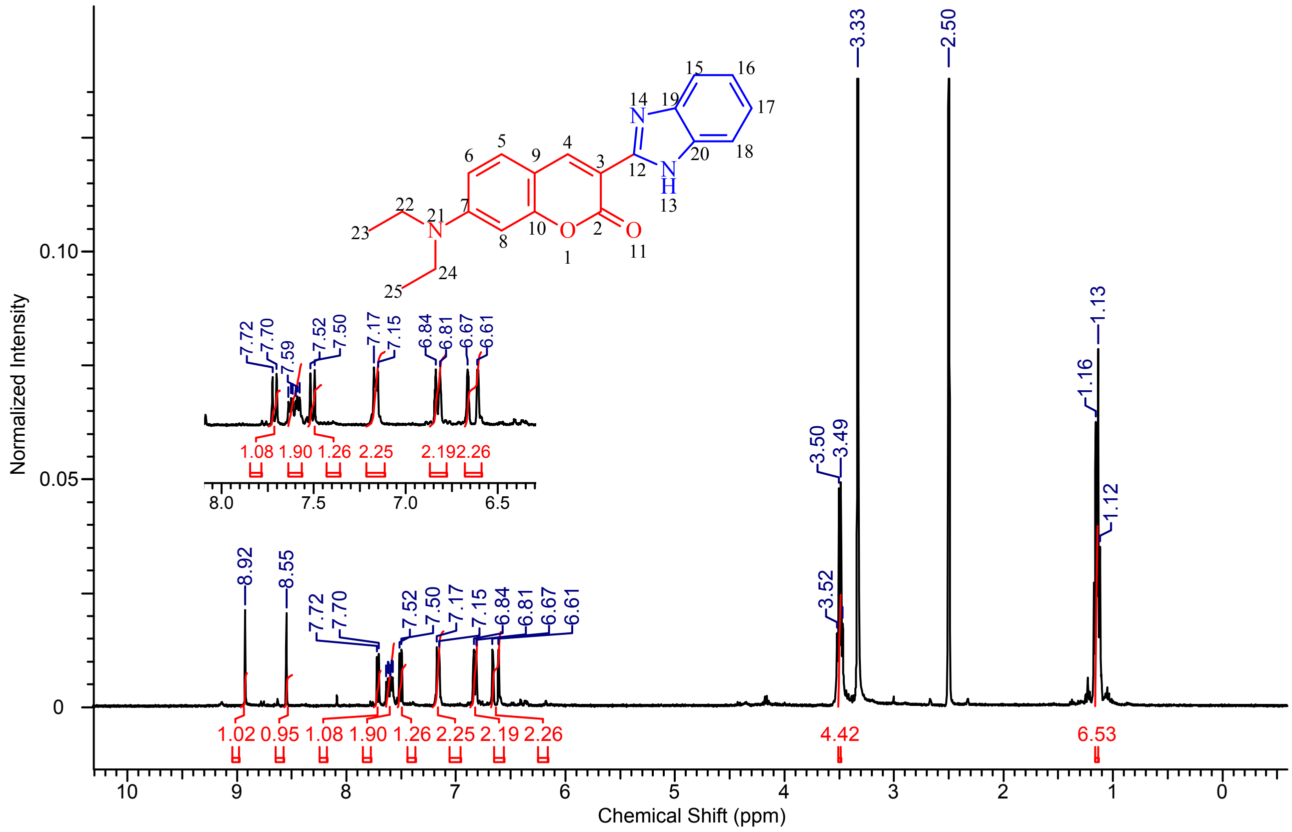

Figure S31. ^{1}H NMR spectrum (500 MHz, DMSO-*d6*) of compound **33**.

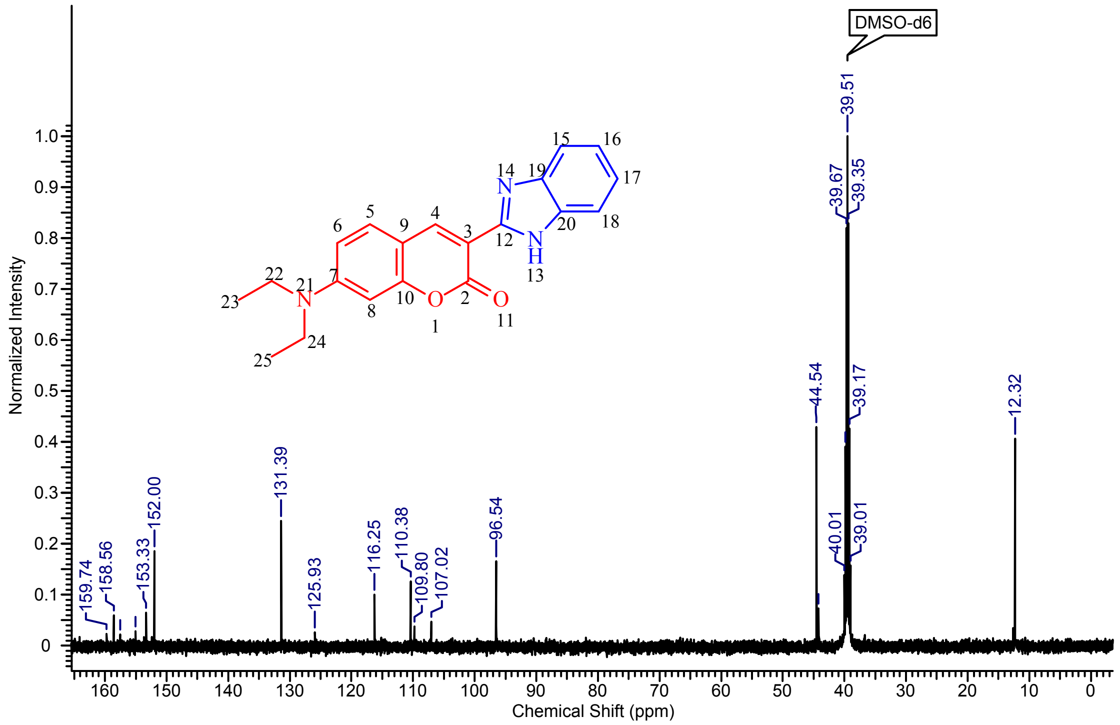

Figure S32. $^{13}C\{^{1}H\}$ NMR spectrum (125 MHz, DMSO-*d6*) of compound **33**.

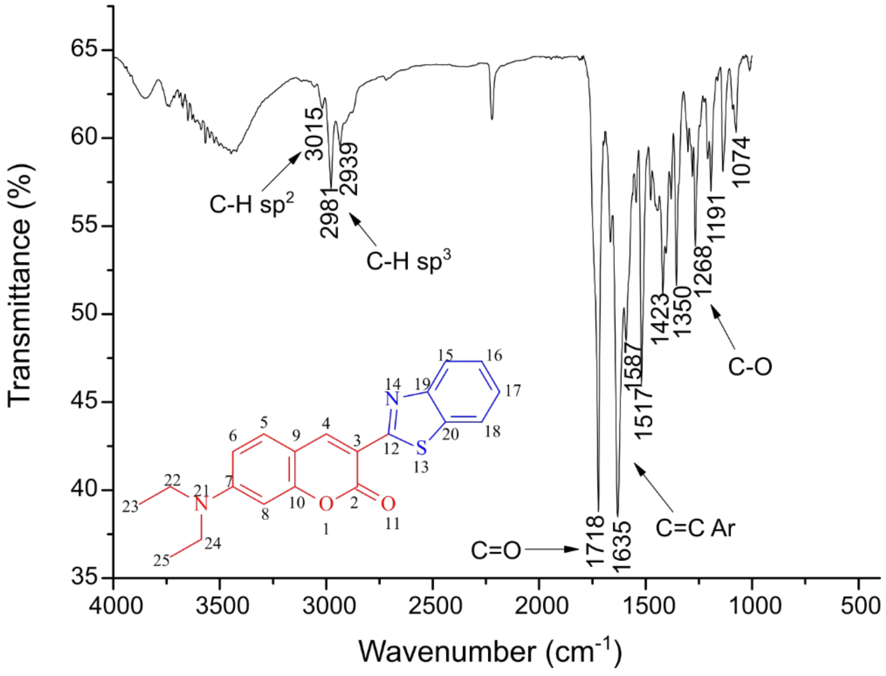

Figure S33. FTIR (KBr) spectrum of compound **34**.

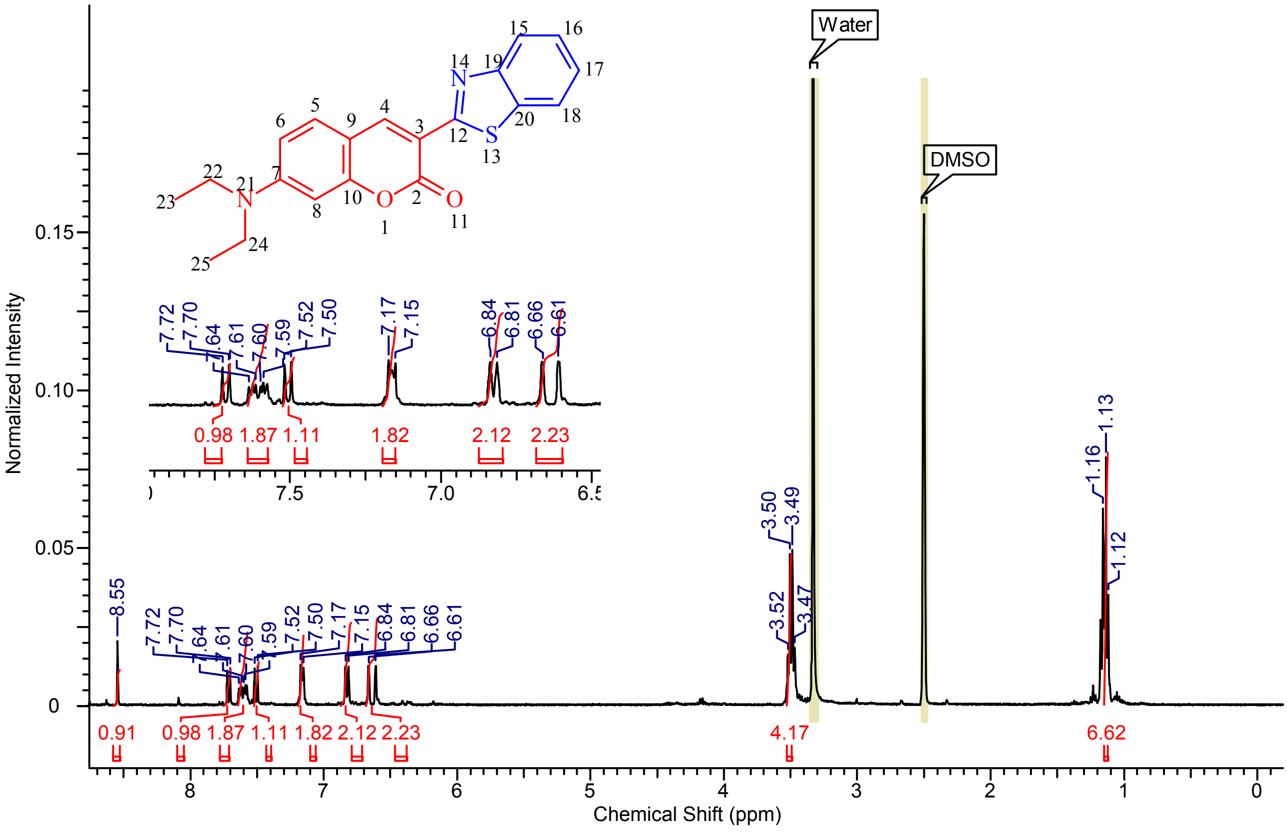

Figure S34. ^{1}H NMR spectrum (500 MHz, DMSO-*d*6) of compound **34**.

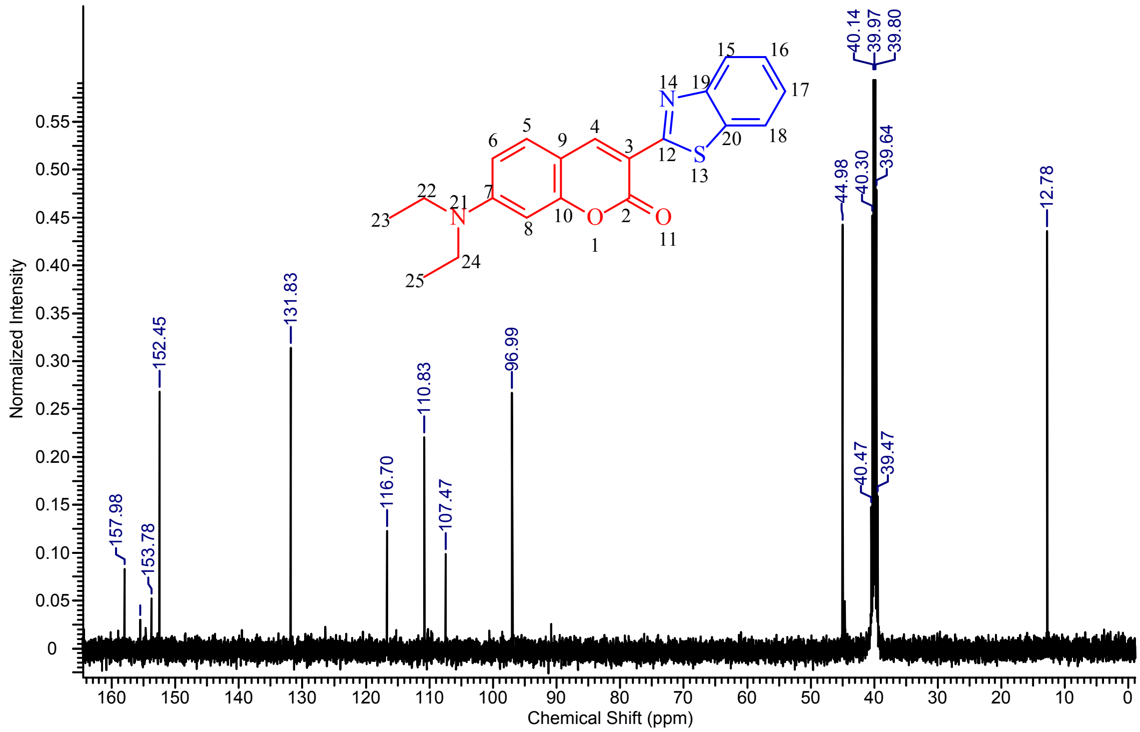

Figure S35. ^{13}C NMR spectrum (125 MHz, DMSO-*d*6) of compound **34**.

SM1 – UV-Vis Absorbance and Fluorescence Emission Spectra

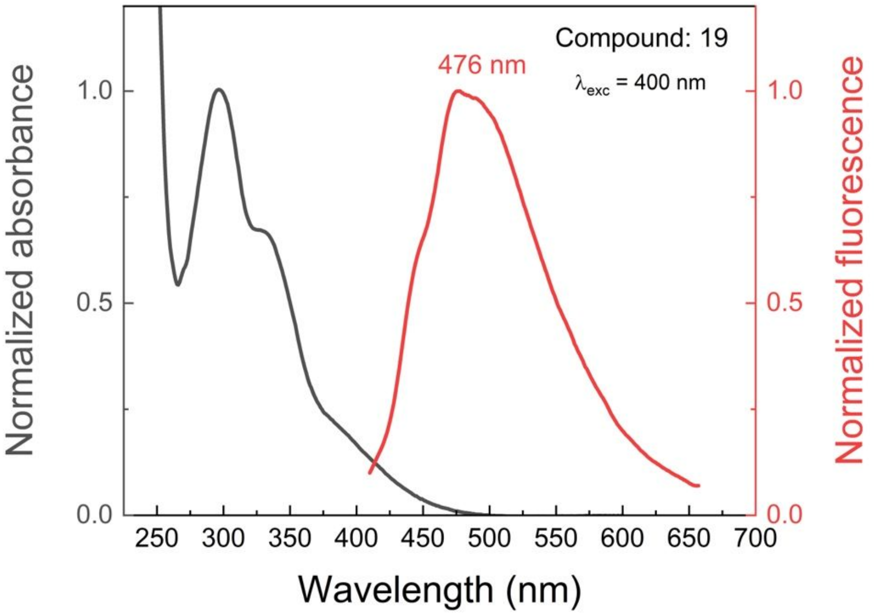

Figure S36. Normalized absorbance and fluorescence emission spectra of compound **19** in DMSO. The absorbance spectrum (black curve) shows a maximum at 297 nm, while the fluorescence emission spectrum (red curve) exhibits a maximum at 476 nm upon excitation at 400 nm.

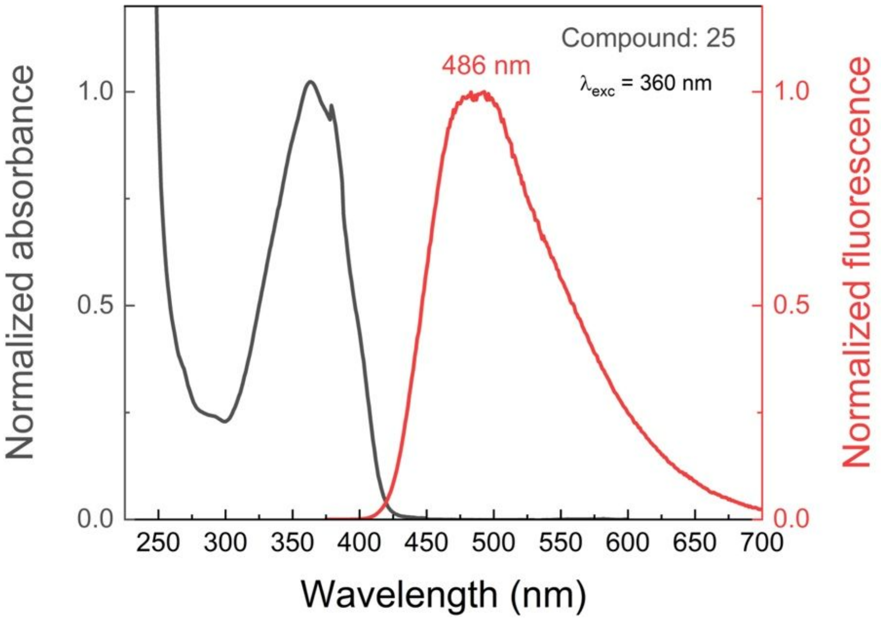

Figure S37. Normalized absorbance and fluorescence emission spectra of compound **25** in DMSO. The absorbance spectrum (black curve) shows a maximum at 365 nm, while the fluorescence emission spectrum (red curve) exhibits a maximum at 486 nm upon excitation at 360 nm.

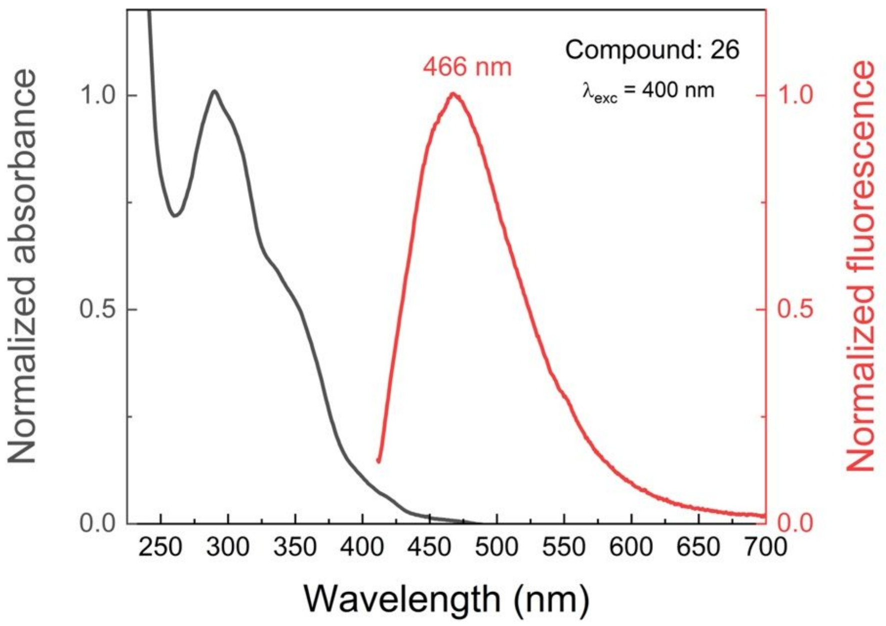

Figure S38. Normalized absorbance and fluorescence emission spectra of compound **26** in Chloroform. The absorbance spectrum (black curve) shows a maximum at 290 nm, while the fluorescence emission spectrum (red curve) exhibits a maximum at 466 nm upon excitation at 400 nm.

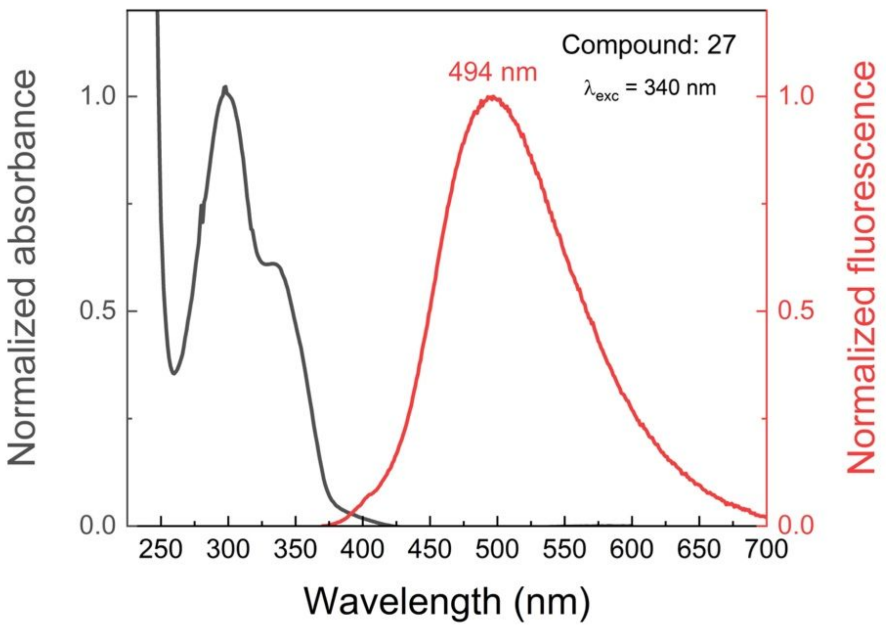

Figure S39. Normalized absorbance and fluorescence emission spectra of compound **27** in DMSO. The absorbance spectrum (black curve) shows a maximum at 298 nm, while the fluorescence emission spectrum (red curve) exhibits a maximum at 494 nm upon excitation at 340 nm.

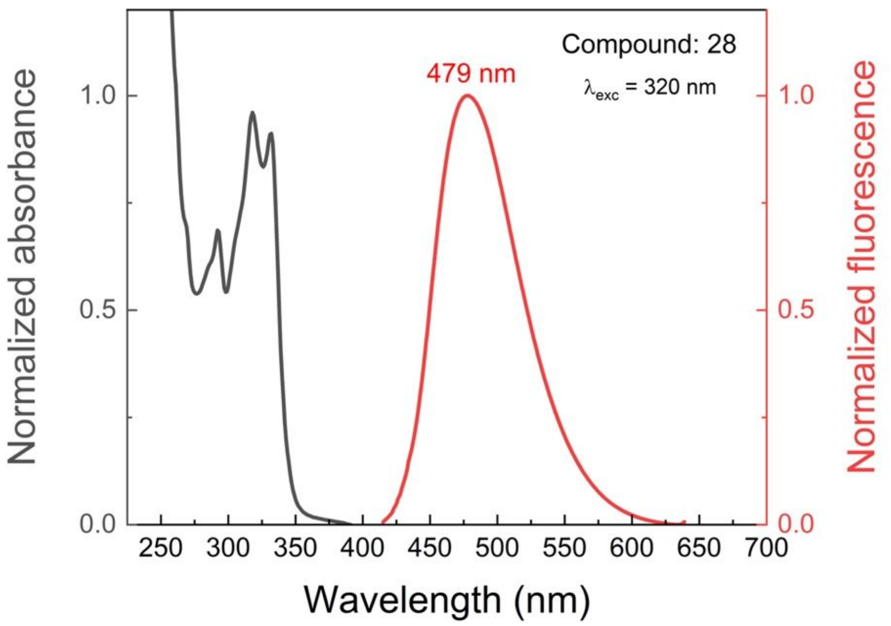

Figure S40. Normalized absorbance and fluorescence emission spectra of compound **28** in DMSO. The absorbance spectrum (black curve) shows a maximum at 318 nm, while the fluorescence emission spectrum (red curve) exhibits a maximum at 479 nm upon excitation at 320 nm.

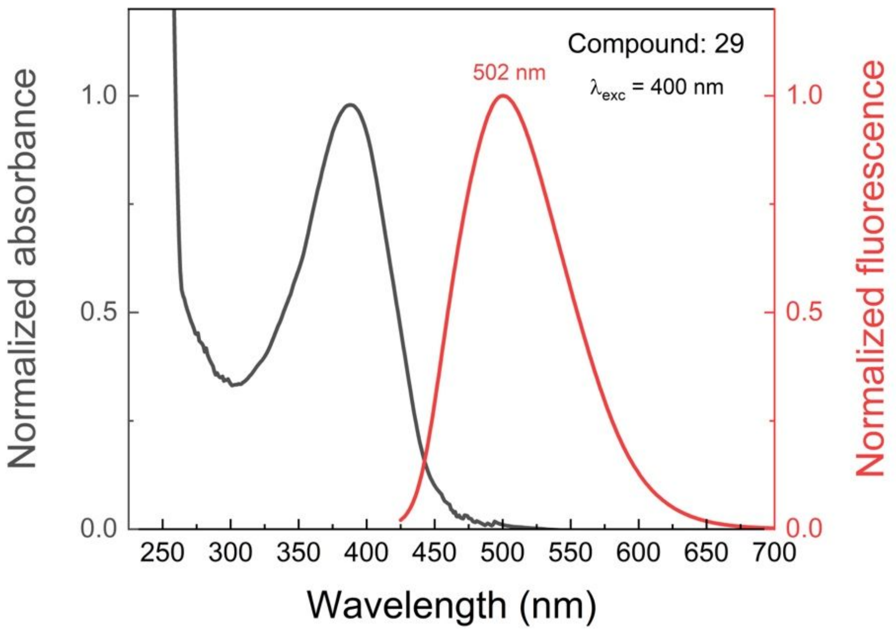

Figure S41. Normalized absorbance and fluorescence emission spectra of compound **29** in DMSO. The absorbance spectrum (black curve) shows a maximum at 388 nm, while the fluorescence emission spectrum (red curve) exhibits a maximum at 502 nm upon excitation at 400 nm.

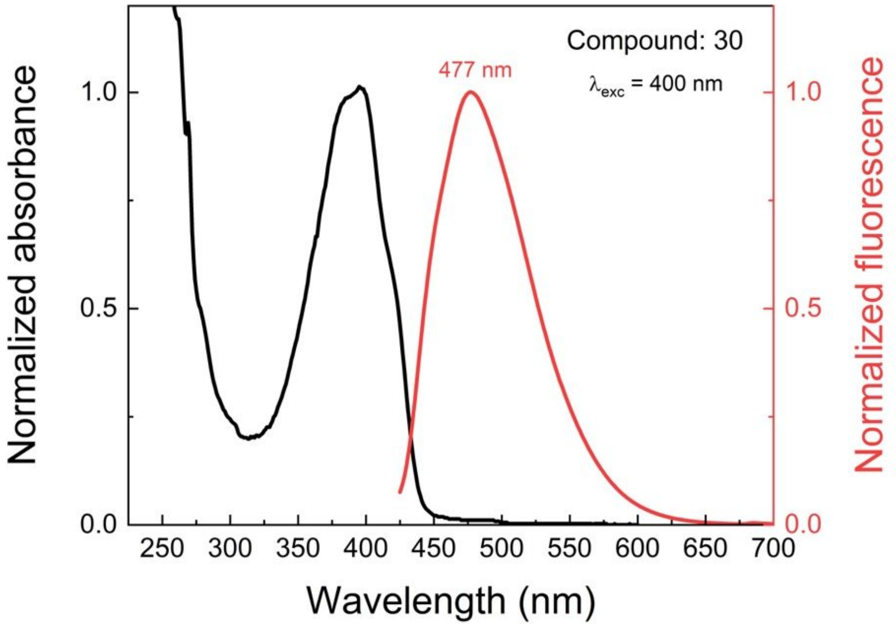

Figure S42. Normalized absorbance and fluorescence emission spectra of compound **30** in Chloroform. The absorbance spectrum (black curve) shows a maximum at 395 nm, while the fluorescence emission spectrum (red curve) exhibits a maximum at 477 nm upon excitation at 400 nm.

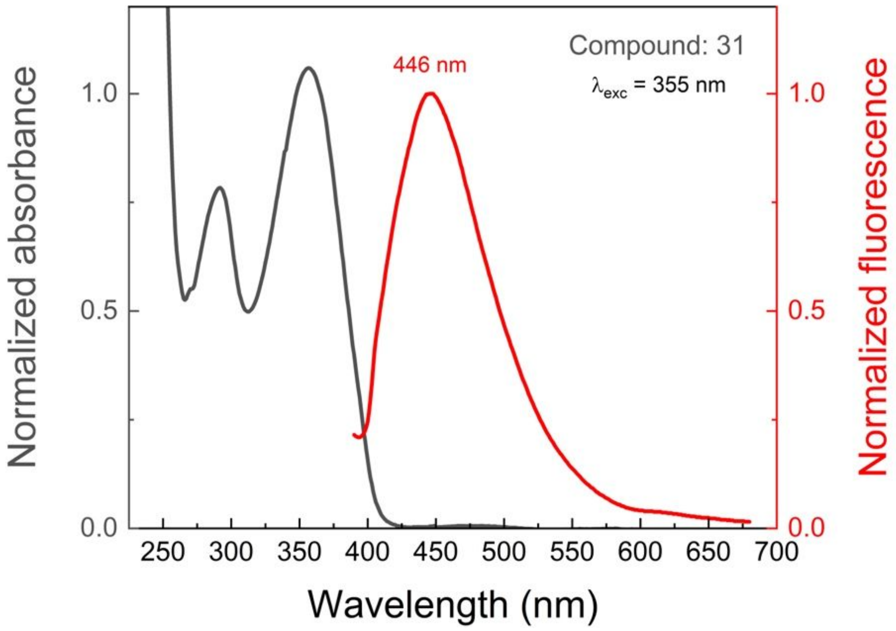

Figure S43. Normalized absorbance and fluorescence emission spectra of compound **31** in Chloroform. The absorbance spectrum (black curve) shows a maximum at 357 nm, while the fluorescence emission spectrum (red curve) exhibits a maximum at 446 nm upon excitation at 355 nm.

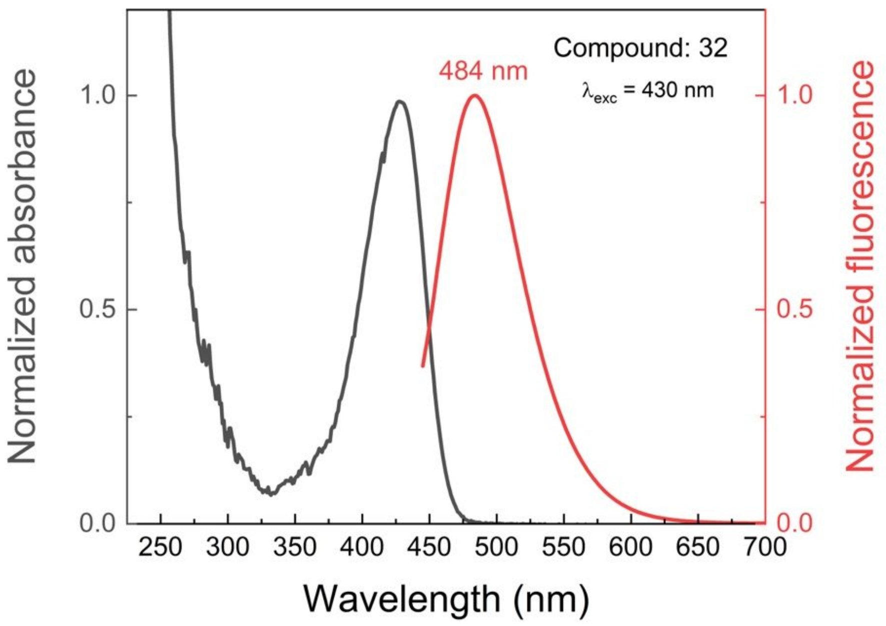

Figure S44. Normalized absorbance and fluorescence emission spectra of compound **32** in DMSO. The absorbance spectrum (black curve) shows a maximum at 429 nm, while the fluorescence emission spectrum (red curve) exhibits a maximum at 484 nm upon excitation at 430 nm.

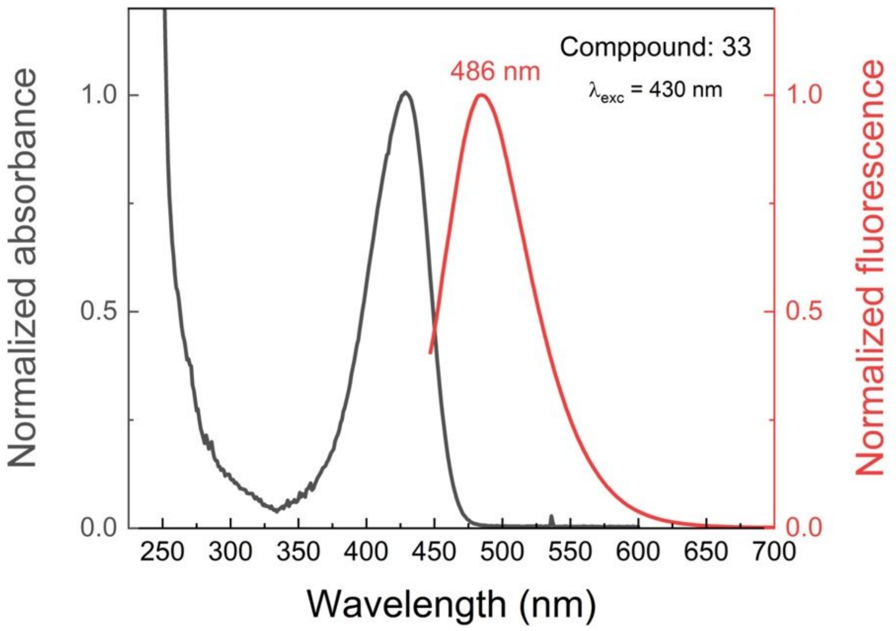

Figure S45. Normalized absorbance and fluorescence emission spectra of compound **33** in DMSO. The absorbance spectrum (black curve) shows a maximum at 429 nm, while the fluorescence emission spectrum (red curve) exhibits a maximum at 486 nm upon excitation at 400 nm.

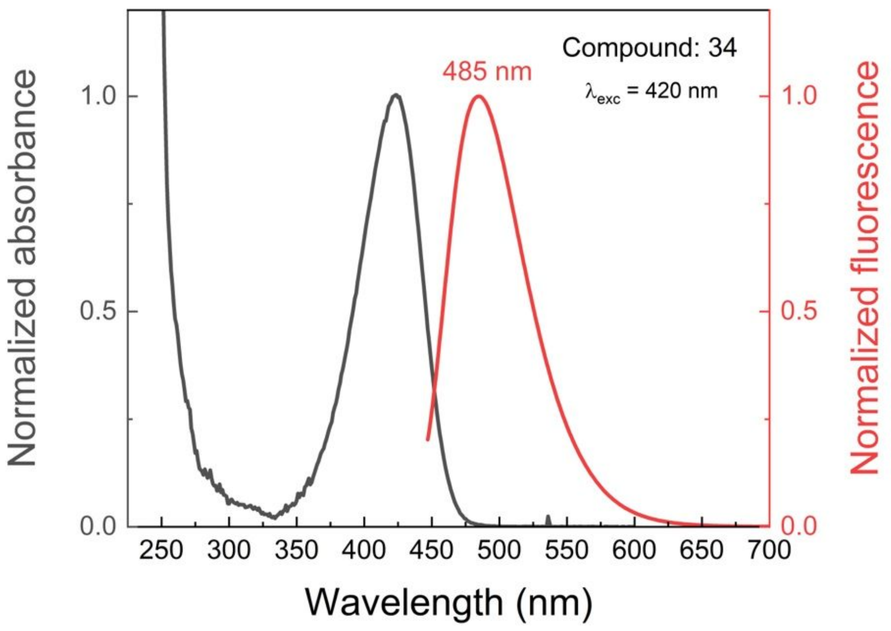

Figure S46. Normalized absorbance and fluorescence emission spectra of compound **34** in DMSO The absorbance spectrum (black curve) shows a maximum at 423 nm, while the fluorescence emission spectrum (red curve) exhibits a maximum at 485 nm upon excitation at 420 nm.

SM1 – Optimized Cartesian Coordinates (Å) of Compounds

Table S1. Optimized Cartesian coordinates (Å) of compound **19** calculated at the M06-2X/6-311++G(d,p) level of theory.

Compound **19**			
C	-4.4276500	1.0871780	-0.0002260
C	-5.5023210	0.2155070	-0.0000230
C	-5.3204120	-1.1818890	-0.0001330
C	-4.0584840	-1.7459420	-0.0000370
N	-1.6082720	-1.1702360	0.0001810
C	-2.9559320	-0.8832550	0.0000990
C	-3.1562110	0.5126100	-0.0000160
N	-1.8909860	1.0493260	-0.0000390
C	-1.0137640	-0.0019060	0.0001260
O	0.3853150	2.5435230	0.0001160
O	2.3763990	1.6111650	0.0000150
C	1.0155200	1.5180290	0.0000590
C	0.4420360	0.1641350	0.0001080
C	1.2553780	-0.9161670	0.0000420
C	3.5792490	-1.8492470	-0.0000270
C	2.6827900	-0.7692790	-0.0000310
C	3.2013320	0.5282200	0.0000530
C	4.5707860	0.7676310	-0.0000640
C	5.4346440	-0.3148450	-0.0001140
C	4.9423250	-1.6254640	-0.0000800
H	-4.5688130	2.1608600	-0.0004410
H	-6.5088970	0.6157590	0.0001650
H	-6.1931820	-1.8236960	0.0000640
H	-3.9109850	-2.8187300	0.0000340
H	-1.6031990	2.0187400	-0.0000350
H	0.8085760	-1.9055690	0.0001570
H	3.1816780	-2.8578590	0.0000380
H	4.9263240	1.7900310	-0.0001510
H	6.5038140	-0.1412150	-0.0002760
H	5.6300660	-2.4613640	-0.0000110

Table S2. Optimized Cartesian coordinates (Å) of compound **25** calculated at the M06-2X/6-311++G(d,p) level of theory.

Compound **25**			
C	-4.4276500	1.0871780	-0.0002260
C	-5.5023210	0.2155070	-0.0000230
C	-5.3204120	-1.1818890	-0.0001330
C	-4.0584840	-1.7459420	-0.0000370
N	-1.6082720	-1.1702360	0.0001810
C	-2.9559320	-0.8832550	0.0000990
C	-3.1562110	0.5126100	-0.0000160
N	-1.8909860	1.0493260	-0.0000390
C	-1.0137640	-0.0019060	0.0001260
O	0.3853150	2.5435230	0.0001160
O	2.3763990	1.6111650	0.0000150
C	1.0155200	1.5180290	0.0000590
C	0.4420360	0.1641350	0.0001080
C	1.2553780	-0.9161670	0.0000420
C	3.5792490	-1.8492470	-0.0000270
C	2.6827900	-0.7692790	-0.0000310
C	3.2013320	0.5282200	0.0000530
C	4.5707860	0.7676310	-0.0000640
C	5.4346440	-0.3148450	-0.0001140
C	4.9423250	-1.6254640	-0.0000800
H	-4.5688130	2.1608600	-0.0004410
H	-6.5088970	0.6157590	0.0001650
H	-6.1931820	-1.8236960	0.0000640
H	-3.9109850	-2.8187300	0.0000340
H	-1.6031990	2.0187400	-0.0000350
H	0.8085760	-1.9055690	0.0001570
H	3.1816780	-2.8578590	0.0000380
H	4.9263240	1.7900310	-0.0001510
H	6.5038140	-0.1412150	-0.0002760
H	5.6300660	-2.4613640	-0.0000110

Table S3. Optimized Cartesian coordinates (Å) of compound **26** calculated at the M06-2X/6-311++G(d,p) level of theory.

Compound **26**			
C	-4.0281830	0.4272870	1.2483800
C	-4.9722870	1.0904030	0.4634230
C	-4.7879050	1.1989080	-0.9096040
C	-3.6583010	0.6566900	-1.5212680
N	-1.5446520	-0.5944310	-1.2296250
C	-2.7228230	-0.0089100	-0.7432610
C	-2.9140700	-0.1195740	0.6354680
S	-1.6173550	-1.0299420	1.4142780
C	-0.7140630	-1.2423560	-0.2120960
O	-0.3854660	1.6033030	0.1406180
O	1.8152560	1.5775470	0.0926990
C	0.5961360	0.9333470	0.0467220
C	0.6297500	-0.5400170	-0.1154430
C	1.8166890	-1.1736830	-0.1670970
C	4.3236750	-1.0737860	-0.1341640
C	3.0666170	-0.4563600	-0.0851330
C	3.0066630	0.9319500	0.0414120
C	4.1619460	1.7040320	0.1188600
C	5.3931740	1.0721600	0.0691680
C	5.4793350	-0.3185370	-0.0575470
H	-4.1652810	0.3382950	2.3192770
H	-5.8471620	1.5244680	0.9309750
H	-5.5199190	1.7215200	-1.5131530
H	-3.4967660	0.7645490	-2.5875630
H	1.8675060	-2.2513290	-0.2796310
H	4.3744960	-2.1528010	-0.2331410
H	4.0682160	2.7781560	0.2165720
H	6.2982310	1.6646480	0.1282380
H	6.4478970	-0.8005530	-0.0963780
H	-1.6724170	-1.1632080	-2.0562430
C	-0.5804210	-2.7344430	-0.5069340
H	-0.0597560	-2.8929280	-1.4568140
H	-1.5796570	-3.1650020	-0.5693080
H	-0.0390480	-3.2551780	0.2842500

Table S4. Optimized Cartesian coordinates (Å) of compound **27** calculated at the M06-2X/6-311++G(d,p) level of theory.

Compound **27**			
C	5.1208430	0.3054590	-0.2374290
C	5.7527770	-0.8701360	0.1211150
C	5.0289450	-2.0097040	0.5320220
C	3.6500980	-2.0023210	0.5947600
N	1.6360180	-0.5424700	0.2130830
C	2.9866460	-0.8220830	0.2357700
C	3.7252490	0.3085920	-0.1743720
N	2.7785040	1.2631340	-0.4470440
H	5.6809480	1.1780580	-0.5495660
H	6.8344930	-0.9189080	0.0884280
H	5.5736990	-2.9055000	0.8036550
H	3.0853160	-2.8702350	0.9117510
H	2.8989460	2.2196490	-0.7474200
O	-1.8682970	2.9612710	0.8118820
O	-3.3025050	1.3141290	0.5010280
C	-2.0306370	1.8504180	0.4163500
C	-0.9932980	0.9538580	-0.1351230
C	-1.2786040	-0.3343790	-0.4258730
C	-2.9691770	-2.1748670	-0.5660920
C	-2.6110730	-0.8511280	-0.2685950
C	-3.5962500	0.0282650	0.1891420
C	-4.9160560	-0.3842890	0.3449260
C	-5.2452040	-1.6956870	0.0462690
C	-4.2748550	-2.5967300	-0.4088850
H	-0.5009500	-0.9944030	-0.7922160
H	-2.2035670	-2.8565260	-0.9197020
H	-5.6492670	0.3277500	0.7015700
H	-6.2695370	-2.0266470	0.1683860
H	-4.5485910	-3.6188880	-0.6372460
C	0.3470710	1.5466220	-0.3880950
C	1.5595060	0.6981270	-0.1953810
O	0.5034250	2.6897130	-0.7470300

Table S5. Optimized Cartesian coordinates (Å) of compound **28** calculated at the M06-2X/6-311++G(d,p) level of theory.

Compound **28**			
C	-4.8123810	1.0767580	0.0003620
C	-5.8850700	0.2020930	0.0003770
C	-5.6989090	-1.1943230	-0.0003610
C	-4.4348450	-1.7545650	-0.0006510
N	-1.9858690	-1.1720460	-0.0002480
C	-3.3348890	-0.8890740	-0.0002150
C	-3.5395680	0.5060690	0.0002090
N	-2.2754960	1.0466290	0.0003600
C	-1.3949320	-0.0018110	0.0000230
O	-0.0007020	2.5497230	0.0000280
O	1.9927490	1.6216880	-0.0004280
C	0.6276540	1.5233360	-0.0001440
C	0.0596370	0.1707550	0.0000360
C	0.8789380	-0.9072770	0.0001400
C	3.2075930	-1.8262570	0.0005030
C	2.3012250	-0.7563550	0.0001630
C	2.8205270	0.5446850	-0.0001360
C	4.1829410	0.7965510	-0.0001840
C	5.0538980	-0.2842160	-0.0000440
C	4.5680610	-1.6012110	0.0002500
H	-4.9565120	2.1500700	0.0005440
H	-6.8927210	0.5996350	0.0009500
H	-6.5696080	-1.8389650	-0.0002870
H	-4.2844230	-2.8269690	-0.0010340
H	-1.9907300	2.0168630	0.0004050
H	0.4343750	-1.8978170	0.0003490
H	2.8226650	-2.8395630	0.0008120
H	4.5572080	1.8113840	-0.0003520
O	6.3776680	-0.0057250	-0.0006800
H	5.2632140	-2.4335100	0.0003090
H	6.8890830	-0.8196480	0.0041820

Table S6. Optimized Cartesian coordinates (Å) of compound **29** calculated at the M06-2X/6-311++G(d,p) level of theory.

Compound **29**			
C	4.4389300	0.9359380	-0.0002880
C	5.5091950	0.0563600	-0.0001710
C	5.2773320	-1.3397930	-0.0000110
C	4.0069620	-1.8795460	0.0001350
N	1.5644810	-1.2538560	0.0003060
C	2.9188990	-0.9979890	0.0000880
C	3.1536380	0.3897830	-0.0001120
N	1.9023820	0.9576320	-0.0000610
C	0.9978770	-0.0718130	0.0001900
O	-0.3395580	2.5101300	-0.0000110
O	-2.3526980	1.6263620	0.0002410
C	-0.9901900	1.4975640	0.0000830
C	-0.4523390	0.1324280	0.0001320
C	-1.2962070	-0.9267920	0.0000800
C	-3.6463830	-1.7923320	-0.0001870
C	-2.7151870	-0.7438840	-0.0000270
C	-3.2045550	0.5684750	0.0000580
C	-4.5608370	0.8519820	0.0000050
C	-5.4566860	-0.2079710	-0.0000670
C	-5.0014520	-1.5357790	-0.0001360
H	4.5982500	2.0082310	-0.0005450
C	6.9277590	0.5664270	-0.0000810
H	6.1370100	-2.0019560	0.0001260
H	3.8431450	-2.9501040	0.0003560
H	1.6390330	1.9337730	-0.0003250
H	-0.8738570	-1.9269700	0.0000970
H	-3.2850100	-2.8143040	-0.0003880
H	-4.9115430	1.8752480	0.0000360
O	-6.7740210	0.1011730	0.0002450
H	-5.7155790	-2.3519010	-0.0001590
H	-7.3041190	-0.7007630	-0.0027730
H	6.9564520	1.6566020	-0.0015620
H	7.4694540	0.2118120	-0.8805900
H	7.4683990	0.2142340	0.8820510

Table S7. Optimized Cartesian coordinates (Å) of compound **30** calculated at the M06-2X/6-311++G(d,p) level of theory.

Compound **30**			
C	-5.0213980	-0.6985820	-0.0007460
C	-5.9079030	0.3639680	-0.0005210
C	-5.4477990	1.6916910	0.0000910
C	-4.0957820	1.9749800	0.0004720
N	-1.8123180	1.0390980	0.0006080
C	-3.1836790	0.9115960	0.0002660
C	-3.6542670	-0.4136560	-0.0003670
S	-2.3097460	-1.5126310	-0.0003950
C	-1.2342950	-0.1198520	0.0003770
O	0.2660130	-2.6127510	0.0006150
O	2.2274120	-1.6087330	0.0004570
C	0.8527560	-1.5690520	0.0004320
C	0.2302800	-0.2393390	0.0002430
C	1.0066740	0.8705080	0.0002290
C	3.2915160	1.8881860	-0.0001230
C	2.4335810	0.7789770	0.0001420
C	3.0075270	-0.4995430	0.0001770
C	4.3801020	-0.6898420	-0.0000440
C	5.2025590	0.4286040	-0.0003300
C	4.6603140	1.7236130	-0.0003930
H	-5.3776300	-1.7213490	-0.0011910
H	-6.9733590	0.1685340	-0.0007820
H	-6.1659080	2.5026520	0.0002760
H	-3.7244900	2.9923420	0.0009380
H	0.5252870	1.8433760	0.0003070
H	2.8624280	2.8836910	-0.0001840
H	4.7987160	-1.6871680	0.0000580
O	6.5372870	0.2092560	-0.0007040
H	5.3187980	2.5852900	-0.0006970
H	7.0115100	1.0453040	0.0009600

Table S8. Optimized Cartesian coordinates (Å) of compound **31** calculated at the M06-2X/6-311++G(d,p) level of theory.

Compound **31**			
C	-5.8967380	0.4195180	0.0000050
C	-6.7506190	-0.6689630	0.0001890
C	-6.2628810	-1.9914590	0.0000280
C	-4.9074520	-2.2611610	-0.0000970
N	-2.6466790	-1.1557450	-0.0002290
C	-4.0241460	-1.1749390	0.0000010
C	-4.5290270	0.1415240	0.0000450
N	-3.4144600	0.9459170	0.0000650
C	-2.3260340	0.1149930	-0.0002140
O	-1.5150540	2.9043150	-0.0001440
O	0.6316250	2.4284130	0.0000430
C	-0.6775190	2.0416630	-0.0000430
C	-0.9415060	0.5942620	-0.0001170
C	0.0860850	-0.2844140	-0.0000770
C	2.5539680	-0.6922650	0.0000540
C	1.4480820	0.1718720	0.0000130
C	1.6698210	1.5506120	0.0000840
C	2.9548170	2.0807860	0.0000540
C	4.0377780	1.2195980	0.0000250
C	3.8278730	-0.1630220	0.0000300
H	-6.2725850	1.4351070	0.0001120
H	-7.8208710	-0.5018680	0.0006560
H	-6.9709500	-2.8111450	0.0004310
H	-4.5254850	-3.2744000	-0.0002740
H	-3.3517860	1.9548820	0.0004040
H	-0.1359230	-1.3469280	-0.0000010
H	2.3961190	-1.7637480	0.0000430
H	3.0842000	3.1553880	0.0000520
H	5.0466010	1.6111960	-0.0000020
Br	5.3277450	-1.3212330	0.0000190

Table S9. Optimized Cartesian coordinates (Å) of compound **32** calculated at the M06-2X/6-311++G(d,p) level of theory.

Compound **32**			
C	-5.9777080	-0.0457410	0.0000800
C	-6.5967510	-1.2828670	0.0000010
C	-5.8458400	-2.4714050	-0.0001080
C	-4.4655540	-2.4389300	-0.0001270
N	-2.4577430	-1.0060950	0.0000180
C	-3.8215040	-1.1947080	-0.0000300
C	-4.5814420	-0.0111980	0.0000540
S	-3.5228020	1.3643970	0.0001140
C	-2.1591590	0.2535770	0.0000530
O	-1.2565070	3.0172040	0.0000420
O	0.8804480	2.4795410	0.0000070
C	-0.4474120	2.1356260	0.0000200
C	-0.7565780	0.6962230	0.0000160
C	0.2438210	-0.2135090	-0.0000730
C	2.6943520	-0.7049550	-0.0001230
C	1.6199650	0.1978040	-0.0001440
C	1.8869830	1.5688690	-0.0001230
C	3.1906650	2.0526990	-0.0001650
C	4.2425500	1.1540060	-0.0000850
C	3.9858950	-0.2207870	-0.0000550
H	-6.5585460	0.8683410	0.0001540
H	-7.6786810	-1.3359920	0.0000240
H	-6.3612880	-3.4240120	-0.0001370
H	-3.8710660	-3.3441930	-0.0001640
H	-0.0073020	-1.2691780	-0.0001100
H	2.4992300	-1.7703740	-0.0000630
H	3.3577770	3.1220520	-0.0002700
H	5.2642870	1.5106570	-0.0001450
Br	5.4474110	-1.4276330	0.0000920

Table S10. Optimized Cartesian coordinates (Å) of compound 33 calculated at the M06-2X/6-311++G(d,p) level of theory.

Compound 33			
C	6.2395820	1.0568050	-0.0334060
C	7.3099990	0.1789750	-0.0665980
C	7.1184410	-1.2158130	-0.0934930
C	5.8515940	-1.7714480	-0.0883400
N	3.4045110	-1.1816900	-0.0407720
C	4.7547440	-0.9032990	-0.0547650
C	4.9651530	0.4909660	-0.0276380
N	3.7030200	1.0354470	0.0028900
C	2.8175070	-0.0092990	-0.0062290
O	1.4423710	2.5522070	0.0926360
O	-0.5556670	1.6338580	0.0993150
C	0.8089900	1.5264150	0.0730590
C	1.3655360	0.1742040	0.0228570
C	0.5345040	-0.8995730	0.0072890
C	-1.8116040	-1.7914660	0.0393060
C	-0.8801030	-0.7385830	0.0386590
C	-1.3928660	0.5617320	0.0794010
C	-2.7499390	0.8337740	0.1206130
C	-3.6740700	-0.2236020	0.1069900
C	-3.1630140	-1.5520620	0.0711110
H	6.3873510	2.1295050	-0.0126830
H	8.3188840	0.5734510	-0.0718840
H	7.9863690	-1.8638200	-0.1179120
H	5.6975100	-2.8432030	-0.1085320
H	3.4192400	2.0056470	0.0319440
H	0.9724730	-1.8926220	-0.0278000
H	-1.4455110	-2.8116270	0.0021440
H	-3.0449930	1.8693270	0.1972680
N	-5.0329750	-0.0075420	0.1177140
H	-3.8440420	-2.3904910	0.0395520
C	-5.5643420	1.3496250	0.0481510
C	-6.9362850	1.4237710	-0.6135300
C	-5.9202400	-1.0377040	0.6584690
C	-6.5816480	-1.9082080	-0.4075080
H	-5.6026530	1.7990780	1.0516860
H	-4.8804140	1.9472010	-0.5525190
H	-7.2487320	2.4681250	-0.6688740
H	-6.8926620	1.0242360	-1.6283550
H	-7.7031340	0.8811200	-0.0588660
H	-5.3530810	-1.6569870	1.3570370
H	-6.6855240	-0.5364580	1.2577720
H	-7.1961790	-2.6801230	0.0618110
H	-7.2194410	-1.3094880	-1.0587260
H	-5.8326210	-2.3961400	-1.0338780

Table S11. Optimized Cartesian coordinates (Å) of compound **34** calculated at the M06-2X/6-311++G(d,p) level of theory.

Compound **34**			
C	-6.4240680	-0.6215530	-0.0591100
C	-7.2884170	0.4591270	-0.0960810
C	-6.7999090	1.7761250	-0.1105410
C	-5.4419180	2.0304360	-0.0880740
N	-3.1792320	1.0487370	-0.0249150
C	-4.5527090	0.9487790	-0.0506380
C	-5.0516540	-0.3660760	-0.0365720
S	-3.7299380	-1.4925390	0.0110800
C	-2.6238500	-0.1212820	0.0076090
O	-1.1811030	-2.6478210	0.0888290
O	0.8003330	-1.6837760	0.0958900
C	-0.5735210	-1.6139010	0.0756970
C	-1.1650340	-0.2746820	0.0384960
C	-0.3599970	0.8197990	0.0326420
C	1.9574460	1.7761560	0.0707130
C	1.0575200	0.6962330	0.0601040
C	1.6062230	-0.5906470	0.0844890
C	2.9710610	-0.8215860	0.1173320
C	3.8641480	0.2628430	0.1127720
C	3.3152760	1.5769220	0.0958860
H	-6.8017570	-1.6365850	-0.0478330
H	-8.3575420	0.2857240	-0.1140400
H	-7.5001620	2.6021210	-0.1398900
H	-5.0491280	3.0396580	-0.0989140
H	-0.8195800	1.8029750	0.0074290
H	1.5614680	2.7855500	0.0465850
H	3.2972490	-1.8486070	0.1811390
N	5.2280200	0.0851390	0.1148860
H	3.9709810	2.4356110	0.0728350
C	5.7984610	-1.2549640	0.0221370
C	7.1662820	-1.2796570	-0.6516560
C	6.0901700	1.1350820	0.6574540
C	6.7142490	2.0362660	-0.4057210
H	5.8582380	-1.7164640	1.0189990
H	5.1273770	-1.8642090	-0.5811920
H	7.5079360	-2.3135920	-0.7256070
H	7.1024640	-0.8663930	-1.6599290
H	7.9221470	-0.7236760	-0.0951020
H	5.5125010	1.7300100	1.3684960
H	6.8760890	0.6503210	1.2433610
H	7.3068890	2.8236750	0.0660350
H	7.3657970	1.4648880	-1.0676460
H	5.9441200	2.5047210	-1.0211870

REFERENCES

(1) Kalalbandi, V. K. A.; Seetharamappa, J. POCl3-Mediated Synthesis of 2-Substituted Benzimidazolyl-Coumarin, Benzimidazolyl-Indole, and Styrylbenzimidazole Derivatives. *Synth Commun* **2016**, *46* (7), 626–635. https://doi.org/10.1080/00397911.2016.1160412.

(2) Khoobi, M.; Ramazani, A.; Foroumadi, A. R.; Hamadi, H.; Hojjati, Z.; Shafiee, A. Efficient Microwave-Assisted Synthesis of 3-Benzothiazolo and 3-Benzothiazolino Coumarin Derivatives Catalyzed by Heteropoly Acids. *Journal of the Iranian Chemical Society* **2011**, *8* (4), 1036–1042. https://doi.org/10.1007/BF03246560.

(3) H. Elnagdi, M.; O. Abdallah, S.; M. Ghoneim, K.; M. Ebied, E.; N. Kassab, K. Synthesis of Some Coumarin Derivatives as Potential Laser Dyes. *J Chem Res Synop* **1997**, No. 2, 44–45. https://doi.org/10.1039/A603731C.

(4) Li, C.; Wang, S.; Huang, Y.; Wen, Q.; Wang, L.; Kan, Y. Photoluminescence Properties of a Novel Cyclometalated Iridium(Iii) Complex with Coumarin-Boronate and Its Recognition of Hydrogen Peroxide. *Dalton Transactions* **2014**, *43* (14), 5595–5602. https://doi.org/10.1039/C3DT53498G.

(5) Kovalenko, S. N.; Vasil'ev, M. V; Sorokina, I. V; Chernykh, V. P.; Turov, A. V; Rudnev, S. A. Recyclization of 2-Imino-2H-1-Benzopyrans Using Nucleophilic Reagents 3. Reaction of 2-Iminocoumarin-3-Carboxamides with o-Phenylenediamines and o-Amino(Thio)Phenols. *Chem Heterocycl Compd (N Y)* **1998**, *34* (12), 1412–1415. https://doi.org/10.1007/BF02317811.

(6) Abdel-Aziem, A.; Baaiu, B. S.; Abdelhamid, A. O. Synthesis and Evaluation of Antimicrobial Activity of Some Novel Heterocyclic Compounds from 5-Bromosalicylaldehyde. *J Heterocycl Chem* **2017**, *54* (6), 3471–3480. https://doi.org/https://doi.org/10.1002/jhet.2970.

(7) Nourmohammadian, F.; Gholami, M. D. Microwave-Promoted One-Pot Syntheses of Coumarin Dyes. *Synth Commun* **2010**, *40* (6), 901–909. https://doi.org/10.1080/00397910903026699.

(8) Bhagwat, A. A.; Sekar, N. Fluorescent 7-Substituted Coumarin Dyes: Solvatochromism and NLO Studies. *J Fluoresc* **2019**, *29* (1), 121–135. https://doi.org/10.1007/s10895-018-2316-2.
